# Ruddlesden–Popper Perovskites: Synthesis and Optical Properties for Optoelectronic Applications

**DOI:** 10.1002/advs.201900941

**Published:** 2019-10-16

**Authors:** Xupeng Gao, Xiangtong Zhang, Wenxu Yin, Hua Wang, Yue Hu, Qingbo Zhang, Zhifeng Shi, Vicki L. Colvin, William W. Yu, Yu Zhang

**Affiliations:** ^1^ State Key Laboratory of Integrated Optoelectronics and College of Electronic Science and Engineering Jilin University Changchun 130012 China; ^2^ Department of Chemistry and Physics Louisiana State University Shreveport LA 71115 USA; ^3^ Department of Chemistry Brown University Providence RI 02912 USA; ^4^ Key Laboratory of Materials Physics of Ministry of Education Department of Physics and Engineering Zhengzhou University Zhengzhou 450052 China

**Keywords:** 2D perovskies, light‐emitting diodes, quantum confinement, solar cells

## Abstract

Ruddlesden–Popper perovskites with a formula of (A′)_2_(A)*_n_*_−1_B*_n_*X_3_ *_n_*_+1_ have recently gained widespread interest as candidates for the next generation of optoelectronic devices. The variations of organic cation, metal halide, and the number of layers in the structure lead to the change of crystal structures and properties for different optoelectronic applications. Herein, the different synthetic methods for 2D perovskite crystals and thin films are summarized and compared. The optoelectronic properties and the charge transfer process in the devices are also delved, in particular, for light‐emitting diodes and solar cells.

## Graphical abstract

**Ruddlesden–Popper 2D perovskites with a formula of (A′)_2_(A)*_n_*_−1_B*_n_*X_3_*_n_*_+1_** are excellent materials for the next generation of optoelectronic devices. Their properties can be tuned by selecting different organic amines, metal halides, and number of layers for light‐emitting diodes and solar cells.

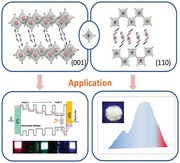

## 1 Introduction

Recently, 3D organometallic halide perovskites with a general formula of ABX_3_ (where A is a monovalent organic ammonium cation such as MA^+^ (CH_3_NH_2_^+^) or FA^+^ (CH(NH_2_)_2_^+^), B is a divalent cation such as Pb^2+^ or Sn^2+^, and X is a monovalent halide anion) have been widely studied in light‐emitting devices (LEDs),^1^ solar cells,^2^ and photodetectors.^3^ These materials have high photoluminescence (PL) purity and broad emission wavelength range,^4^ long‐range charge transport,^5^ and high absorption coefficients.^6^

**Figure 1:**
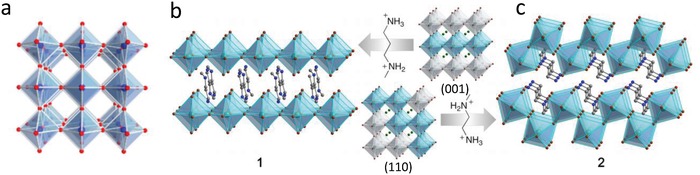
Crystal structures of a) cubic 3D perovskite, b) (001) 2D perovskite (*N*‐MPDA)[PbBr_4_], and c) (110) 2D perovskite (*N*‐MEDA)[PbBr_4_]. *N*‐MPDA = *N*′‐methylpropyl‐1,3‐diammonium; *N*‐MEDA = *N*′‐methylethane‐1,2‐diammonium. a) Reproduced with permission.^9^ Copyright 2018, Annual Reviews. b,c) Adapted with permission.^11^ Copyright 2014, American Chemical Society.

By using the organic–inorganic hybrid lead iodide perovskites as the light absorption materials, the power conversion efficiency (PCE) of solar cells has been greatly improved from 3.8% to 24.2% in the past 10 years.[qv: 2a,7] For stable 3D perovskite structures, a tolerance factor *t* that was proposed by Goldschmidt in 1926 should be in the range of 0.8 ≤ *t* ≤ 1, where *t* = (*R*_A_ + *R*_X_)/$\sqrt{2\;}$(*R*_B_ + *R*_X_), *R*_A_, *R*_B_, and *R*_X_ being respective ionic radii.^8^ Structurally, the metal halide [BX_6_]4^−^ octahedral units are linked together by corner‐sharing halide anions, and the A cations occupy voids within the framework (**Figure** **1**a).^9^ When A was replaced by large organic cations, they do not fit in the interspace between the [BX_6_]4^−^ octahedra, and the network was constrained to form a 2D structure.^10^ Thus, compared with 3D perovskites, 2D perovskite materials exhibit different characteristic properties.

2D and corrugated 2D organometallic halide perovskites are formed by splitting along lattice orientations 〈001〉 and 〈110〉 from 3D perovskites, which are identified as Ruddlesden–Popper perovskites (RPPs) (Figure 1b,c).^11^ The general chemical formula of 2D organometallic halide perovskites is (A′)_2_(A)*_n_*_−1_B*_n_*X_3_ *_n_*_+1_, wherein A′ represents R—NH_3_ or H_3_N—R—NH_3_ (R is a large aliphatic alkyl chain or an aromatic ligand) and acts as an insulating layer to isolate the different inorganic layers that are composed of metal halide octahedral units shared through corner atoms. A represents small cations, such as Cs^+^ and CH_3_NH_3_^+^. B is a divalent metal cation (Pb^2+^ or Sn^2+^) and X refers to halides. Symbol *n* stands for the number of metal halide monolayer sheets in between the insulating A organic layers: *n* = 1, strict 2D structure; *n* = 2–5, quasi‐2D structure; *n* = ∞, conventional 3D structure.^12^ Structurally, for the single ammonium cations, the terminal amine of organic cation interacts with halide of inorganic anion layer through hydrogen bonds, and the adjacent carbon chains of the organic cations are bound together by van der Waals force to form organic layers. For the diammonium cations, the two terminal amines are linked to the inorganic layer through hydrogen bonds.

2D perovskites are considered as active optoelectronic materials for optoelectronic devices due to their large exciton binding energy,^13^ strong quantum confinement effect,^14^ and superior stability to moisture^15^ compared with 3D perovskites. Different selection of cationic ligands, metal halides, and the number of layers of inorganic materials will lead to the change of crystal structure and optical properties of 2D perovskite materials, realizing the bandgap tunability,^16^ narrowband emission,[qv: 15b,17] and broadband emission wavelength.^11^, ^18^ Moreover, these organic ligands have a great influence on the electronic properties of inorganic layer by twisting the soft inorganic framework.^19^ Therefore, it is important to understand the relationship among the material constituents, crystal structures, and optoelectronic properties, so that one can tune the bandgap, transport performance, and charge carrier dynamics and eventually fabricate excellent optoelectronic devices.

The first 2D layered lead halide perovskite (C_9_H_19_NH_3_)_2_PbI_4_ was obtained by Dolzhenko et al. in 1986.^20^ (C_9_H_19_NH_3_)_2_PbI_4_ shows the ability to intercalate with appropriate organic solvents through weak interaction. Another 2D perovskite (C_10_H_21_NH_3_)_2_PbI_4_ was reported by Ishihara et al.,^21^ which is similar to a quantum well (QW) structure where inorganic layers are separated by insulating organic layers. Over the years, 2D organic–inorganic hybrid halide perovskites have been known by researchers, but they have not achieved the same attention as their 3D analogues.

While a number of reviews have been published about 2D perovskites regarding their structure and application in solar cells,^12^, ^22^ we here summarize the recent advances in the synthesis of 2D metal halide perovskites, highlight their unique tunable bandgap, narrowband fluorescence, and white light radiation properties, discuss the charge carriers' transport, and focus more on LEDs. We hope that this review will stimulate more efforts in this field, including materials' preparation and optoelectronic device fabrications.

## 2 Design and Synthesis of 2D Perovskites

Dimensions of perovskites can be controlled by selecting different organic ligands and metal halides. The orientation of the inorganic thin layers is dependent on the geometry and noncovalent bond interaction of spatial cations, and the number of inorganic layers is directly determined by the stoichiometric ratio.[qv: 13a]

### 2.1 2D Perovskite Crystals

#### 2.1.1 Single‐Crystal Growth Methods

Single crystal is the most useful state to analyze the structure and physical properties of materials. 2D single‐crystalline perovskites have been synthesized by a variety of solution methods. Liquid‐phase crystallization involves dissolving the divalent metal halide (MX_2_) and organic amine halide (RNH_2_·HX) at high temperatures in solvent, then mixing them together to start the crystal growth, and later cooling them to room temperature at a certain cooling rate to quench the further growth; (mixed) solvent evaporation is similar to the liquid‐phase crystallization. The single crystals are obtained by evaporating solvent(s) at a relatively slow rate, while the solvent evaporation can be accelerated by adding another solvent; the layered solution method involves dissolving the two reactants in two solvents with certain mutual solubility, and the two solutions have obvious density difference, so as to form a clear interface between the two solutions due to the different solubility and density. With a slow diffusion, large‐sized single crystal is precipitated at the interface. These solution methods have some advantages of stability, low cost, and easy operation. The main solution‐processed methods for synthesizing 2D organic–inorganic hybrid perovskites are summarized in **Table** **1**.

**Table 1:** Summary of the main solution‐processed methods for 2D perovskite single crystals

Perovskite material	Synthesis method
*n* = 1	
(C_4_H_9_NH_3_)_2_PbBr_4_	SE^23^ (DMF), LPC[qv: 17c]
(C_4_H_9_NH_3_)_2_PbI_4_	TPC[qv: 18b,24], LPC[qv: 15a,25]
(C_6_H_5_CH_2_NH_3_)_2_PbCl_4_	LSM^26^
(C_6_H_5_CH_2_NH_3_)_2_PbBr_4_	SE^23^ (DMF), LSM^26^
(C_6_H_5_CH_2_NH_3_)_2_PbI_4_	LSM^26^, ^27^
(C_6_H_5_C_2_H_4_NH_3_)_2_PbCl_4_	LSM,^26^ MSE (DMF/nitromethane)^28^
(C_6_H_5_C_2_H_4_NH_3_)_2_PbI_4_	LSM^26^, ^27^, TPC[qv: 16b,29]
(C_6_H_5_C_4_H_8_NH_3_)_2_PbI_4_	LSM[qv: 27b]
(C_6_H_13_NH_3_)_2_PbI_4_	SE (acetone)^30^
(C_10_H_21_NH_3_)_2_PbCl_4_	LPC^31^
(C_10_H_21_NH_3_)_2_PbBr_4_	MSE (H_2_O/nitromethane)^31^
(C_10_H_21_NH_3_)_2_PbI_4_	MSE (acetone/nitromethane)^31^
[CH(NH_2_)_2_][C(NH_2_)_3_]PbI_4_	LPC^32^
(*N*‐MEDA)[PbBr_4_]	MSE (DMF/acetone)[qv: 11,18a]
Diammonium cation	
(H_3_NC_4_H_8_NH_3_)PbBr_4_	TPC^33^
(H_3_NC_4_H_8_NH_3_)PbI_4_	TPC^33^
(H_3_NC_8_H_16_NH_3_)PbI_4_	TPC^33^
(H_3_NC_10_H_20_NH_3_)PbBr_4_	TPC^33^
(H_3_N(CH_2_)_12_NH_3_)PbI_4_	TPC^33^
(H_3_NC_10_H_6_NH_3_)PbI_4_	TPC^33^
*n* = 2	
(C_4_H_9_NH_3_)_2_(CH_3_NH_3_)Pb_2_I_7_	TPC,[qv: 24a] LPC^25^
(C_6_H_5_C_2_H_4_NH_3_)_2_(CH_3_NH_3_)Pb_2_I_7_	TPC[qv: 16b,29]
(C_6_H_13_NH_3_)_2_(CH_3_NH_3_)Pb_2_I_7_	SE (DMF)^30^
(C*_x_*H_2_ *_x_*_+1_NH_3_)_2_(CH_3_NH_3_)Pb_2_Br_7_ (*x* = 2, 3, 4, and 6)	MSE (DMF/acetone)^34^
*n* = 3	
(C_4_H_9_NH_3_)_2_(CH_3_NH_3_)_2_Pb_3_I_10_	TPC,[qv: 24a] LPC^25^, ^35^
(C_6_H_5_C_2_H_4_NH_3_)_2_(CH_3_NH_3_)_2_Pb_3_I_10_	TPC[qv: 16b,29]
(CH_3_CH_2_NH_3_)_4_Pb_3_Cl_10_	SE[qv: 18c]
(CH_3_CH_2_NH_3_)_4_Pb_3_Br_10_	SE[qv: 18c]
(C_6_H_13_NH_3_)_2_(CH_3_NH_3_)Pb_3_Br_10_	MSE (DMF/acetone)^34^
*n* = 4	
(C_4_H_9_NH_3_)_2_(CH_3_NH_3_)_3_Pb_4_I_13_	TPC[qv: 24a] LPC[qv: 25a,36]
(C_6_H_5_C_2_H_4_NH_3_)_2_(CH_3_NH_3_)_2_Pb_3_I_13_	TPC^29^

LPC = liquid‐phase crystallization; MSE = mixed solvent evaporation; SE = solvent evaporation; TPC = temperature‐programmed crystallization; LSM = layered solution method.

**Figure 2:**
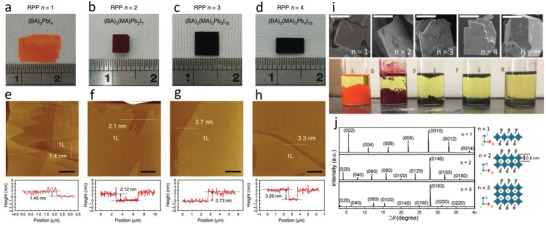
a–d) Photographs of centimeter‐sized RPP single crystals with *n* from 1 to 4. e–h) AFM images and corresponding height profiles along the dotted lines in the images of the monolayer (1L) RPP series (*n*  =  1–4) (scale bars = 4 µm). a–h) Reproduced with permission.[qv: 24a] Copyright 2018, Springer Nature. i) SEM images (top panel) and photographs of the (BA)_2_(MA)*_n_*_−1_Pb*_n_*I_3_ *_n_*_+1_ perovskite crystals (bottom panel) (scale bars = 200 µm). Reproduced with permission.[qv: 25a] Copyright 2016, American Chemical Society. j) XRD patterns by lock‐in coupled θ–2θ scan of the freshly cleaved single crystals of PEA_2_PbI_4_ · (MAPbI_3_)*_n_*_−1_ (*n* = 1, 2, 3) (left), and schematic illustrations of the layered structure and the corresponding orientation of 2D perovskite crystals showing that the thickness of a single perovskite sheet is ≈0.6 nm (right). Adapted with permission.[qv: 16b] Copyright 2017, American Chemical Society.

2D perovskites can be synthesized by enormous alkylammonium cations with different lengths that mainly act as the structural guides to regulate the interlayer spacing among inorganic layers.^37^ Leng et al.[qv: 24a] reported a temperature‐programmed crystallization method to achieve a series of 2D perovskites (BA)_2_(CH_3_NH_3_)*_n_*_−1_Pb*_n_*I_3_ *_n_*_+1_ (*n* = 1, 2, 3, 4) (BA = C_4_H_9_NH_3_^+^). Typically, the uniform solution including varied mass ratios of PbO, BAI, MAI, and HI (containing H_3_PO_2_) was heated to boiling with magnetic stirring. Then, the large‐sized monolayer perovskites could be separated out from the solutions after cooling down from 110 °C to room temperature at a rate of 3 °C h^−1^ (**Figure** **2**a–d). Atomic force microscopy (AFM) images indicate that the monolayer's thickness and the *n* value have a good agreement with the *c*‐axis lattice constants (Figure 2e–h) of mono‐unit cell 2D RPPs. By this method, larger‐size and higher‐quality single crystals of 2D hybrid perovskite structures can be obtained.

In 1996, Mitzi reported the synthesis of BA_2_PbI_4_ by liquid‐phase crystallization.^38^ Lately, Stoumpos et al.[qv: 25a] also synthesized similar Ruddlesden–Popper (BA)_2_(CH_3_NH_3_)*_n_*_−1_Pb*_n_*I_3_ *_n_*_+1_ (*n* = 1, 2, 3, 4) by liquid‐phase crystallization. After adding neutralized base BA into the hot aqueous solution containing HI, H_3_PO_2_, and PbO, followed by a cooling process, the colorful rectangular shape sheets started to crystallize and the color changed from red to black (Figure 2i). The crystallization was completed 2 h later. BA is essential to guide the reaction for accurate control of the stoichiometry.^30^, ^39^ A cleaner and easier operation method was reported to make (C_6_H_13_NH_3_)_2_PbI_4_ monolayer perovskite by slowly evaporating the acetone solvent.^30^ Both (C_6_H_13_NH_3_)_2_(CH_3_NH_3_)Pb_2_Br_7_ and (C_6_H_13_NH_3_)_2_(CH_3_NH_3_)Pb_3_Br_10_ were reported by Tabuchi et al.^34^ by a similar preparation. The precipitation method has the characteristic of easy operation, but is relatively time consuming. A large‐sized 4 × 10 × 0.1 mm^3^ (C_10_H_21_NH_3_)_2_PbI_4_ crystal was obtained through evaporating an acetone/nitromethane solution.^31^ The addition of nitromethane accelerated the growth of single crystal, so that single crystal grew in a shorter time. Using the same method, (C_10_H_21_NH_3_)_2_PbBr_4_ and (C_10_H_21_NH_3_)_2_PbCl_4_ crystals were also prepared, but acetone was replaced by water due to the poor solubility of (C_10_H_21_NH_3_)_2_PbBr_4_ in it.^31^, ^40^

In addition to single ammonium cations, diammonium cations are also introduced to synthesize 2D perovskites. Diammonium cations have an advantage that complex R′ is more liable to form 2D layers and diammonium cations can eliminate van der Waals gaps and directly connect the layers together.[qv: 10b,33] 2D diammonium single‐crystal NH_3_(CH_2_)*_n_*NH_3_PbI_4_ (*n* = 4, 6, 8) perovskites were prepared by the solvent evaporation method.^41^ Although the inorganic layers are slightly distorted by the spatial constraints imposed by the diammonium cations, these perovskites have a typical corner‐sharing structure. Through the crystallography in these 2D materials, the well‐defined cation positions showed slower cation movement and migration than MAPbI_3_, which is capable of overcoming stability problems.

Organic layers containing functional groups have also been adopted to synthesize 2D perovskites and bring new functionality.^42^ By temperature‐controlled crystallization, when the solution cools to room temperature at certain rates, single crystals of 2D hybrid perovskite PEA_2_PbI_4_·(MAPbI_3_)*_n_*_−1_ (*n* = 1, 2, 3, 4) (PEA = C_8_H_9_NH_3_^+^) were prepared.^29^ The thicknesses of the single crystals obtained are between 20 and 100 µm, and the spin coherence lifetime is affected by Rashba splitting and phonon scattering, both depending on the layer thickness. When *n* = 1, the sample has a larger recombination rate constant due to the large exciton binding energy compared with *n* = 2, 3, 4 samples, which is beneficial to light‐emitting applications. Peng et al. believe that the decrease of the level of self‐doping and the decrease of the crystal sizes are the result of the defect‐inhibiting crystallization process by introducing large organic cation PEA.[qv: 16b] The diffraction patterns of PEA_2_PbI_4_·(MAPbI_3_)*_n_*_−1_ were indexed as shown in Figure 2j. They calculated the lattice distance of the first diffraction peaks of different *n* values to realize that the increment is the thickness of the single‐layer PbI_6_ (0.6 nm).

Kamminga et al. used four phenyl alkylammonium cations with different alkyl chains of one to four carbons to prepare single‐crystal perovskites at room temperature by a layered solution technique.[qv: 27a,43] The obtained products have good stability and can be stored in low humidity for several months without damage. It is interesting that two compounds with PMA (C_6_H_5_CH_2_NH_3_^+^) and PEA cations have 2D perovskite structure where inorganic layers are linked by corner‐sharing PbI_6_ octahedra isolated by bilayers of organic cations.[qv: 42c,44] However, with longer carbon chains, the 1D perovskites with inorganic layers consisting of corner‐sharing and face‐sharing PbI_6_ octahedra are obtained. Subsequently, Ye's group synthesized (PMA)_2_PbI_4_, (PEA)_2_PbI_4_, and (PBA)_2_PbI_4_ (PBA = C_6_H_5_(CH_2_)_4_NH_3_^+^) perovskites by the same method. The source of fluorescence and the behavior of excitons were confirmed by experiments, and the quantum confinement effect caused by the structural reorganization was demonstrated by calculation.[qv: 27b] A series of phenyl‐ and naphthyl‐containing amine 2D perovskites with non‐centrosymmetric structures were achieved through a simple and high‐yielding liquid‐phase crystallization.^26^ These 2D perovskites possess broad white fluorescence emission in the long‐wavelength region resulting from the inorganic layer distortion induced by the introduction of large organic cations.

In addition to lead 2D perovskites, many efforts have been applied for the synthesis of non‐lead 2D perovskites.^45^ In 1994, Mitzi prepared 2D Sn‐based perovskite (BA)_2_(MA)*_n_*_−1_Sn*_n_*I_3_ *_n_*_+1_ by liquid‐phase crystallization in the argon atmosphere to prevent oxidation. Unlike oxide perovskites, which were synthesized at high temperatures, these materials could be produced at lower temperatures. When the precursor solution containing SnI_2_, C_4_H_9_NH_3_I, and CH_3_NH_3_I was cooled down at a rate of 2–5 °C h^−1^ from 90 to 10 °C, the plate‐like products were obtained. When *n* = 3, the orthorhombic structure was obtained.^46^ Later, Mitzi reported a layered Ge‐based perovskite BA_2_GeI_4_,^38^ whose crystal structure and optical properties were studied. Recently, Han's group reported a 2D lead‐free (PEA)_2_GeI_4_ perovskite, prepared by the liquid‐phase crystallization, which was precipitated by cooling HI and H_3_PO_2_ mixed solution containing stoichiometric GeO_2_ and PEAI.^47^ Its direct bandgap is 2.12 eV. They also found that the introduction of PEA cation for the layered structure could actually improve the perovskite stability.

#### 2.1.2 Colloidal Synthesis

Solution‐processed methods that strongly rely on stoichiometric ratios are simple to operate, but take longer time to crystallize. Colloidal synthesis as a mature method has previously been widely used to synthesize inorganic quantum dots (QDs).^48^ Schmidt et al. first synthesized MAPbBr_3_ QD colloidal dispersions, which resulted in high luminescence and good dispersion due to the surface ligand capping.^49^ Recently, a number of 2D perovskites have been obtained by this method.

**Figure 3:**
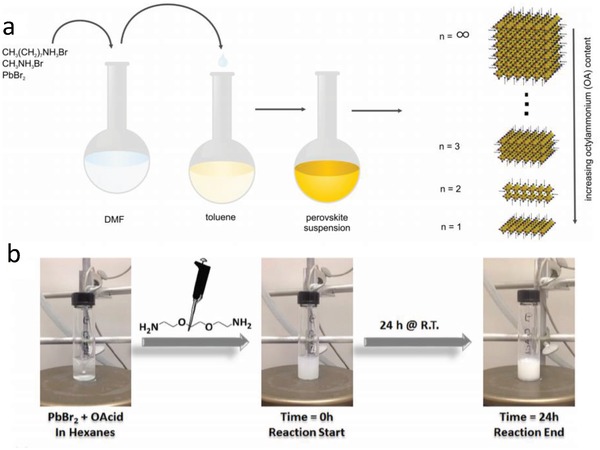
a) Raw materials and synthetic route. Reproduced with permission.^50^ Copyright 2015, American Chemical Society. b) One‐pot synthetic procedure and its reagents and products. Reproduced with permission.^53^ Copyright 2016, Wiley‐VCH.

Feldmann's group^50^ realized 3D to 2D conversion of halide perovskites with varied thickness through regulating the proportion of octylamine by modifying Schmidt's method (**Figure** **3**a). First, MABr and OABr (OA = octylamine) were obtained by adding HBr to a solution of methylamine and octylamine in ethanol, respectively. Excess acid was used to ensure that the amines were completely protonated, and a rotary evaporator was utilized to help the crystallization of ammonium salts. Then, PbBr_2_, OABr, and MABr were mixed in dimethylformamide (DMF) with desired proportions and underwent heating to form a uniform solution. Finally, under vigorous agitation, this precursor solution was dropwise added into toluene. The product was precipitated by centrifugation and was redispersed in toluene. As the ratio of OA increases, the thickness of the nanosheet gradually shrinks until reaching a monolayer.

In order to understand the effect of ligands in the formation of 2D perovskites, a series of (C_8_H_17_NH_3_)_2_(CH_3_NH_3_)_2_Pb_3_(I*_x_*Br_1−_ *_x_*)_10_ 2D perovskite nanorods were prepared. On one hand, sufficient octylamine can stabilize the perovskite surface; on the other hand, enough oleic acid can ensure the control of morphology through the molar ratio of OAI/OAc (OAc = oleic acid).^51^ Weidman et al. achieved fully tunable colloidal 2D perovskite L_2_[ABX_3_]*_n_*_−1_BX_4_ through different organic cation, metal, and halide components. They found that the changes of absorption and emission wavelengths were the result of the change of B or X, while A species can greatly affect the photoluminescence quantum yield (PLQY) and stability.^52^

Zhang's group selected toluene as a solvent and obtained (PEA)_2_PbX_4_ perovskite nanosheets. Then, they studied the effect of three solvents (chlorobenzene, chloroform, and dichloromethane) on the crystallization process.[qv: 15d] The results proved that lateral size of 2D perovskites is tunable through changing solvents. More importantly, single‐layer (PEA)_2_PbI_4_ is more stable under light irradiation and ambient conditions than the conventional 3D MAPbI_3_ QDs. To synthesize corrugated (EDBE)PbBr_4_ halide perovskite (EDBE = 2,2′‐(ethylenedioxy)bis(ethylammonium)), PbBr_2_ was dissolved in nonpolar hexane containing octanoic acid; later, a turbid solution formed by injecting EDBE. The reaction solution was strongly stirred continuously for 24 h until a white colloidal solution was achieved (Figure 3b).^53^ White LEDs were then obtained from (EDBE)PbBr_4_ aroused by a 365 nm UV LED chip.

Hot injection is frequently used to prepare conventional inorganic QDs^54^ and perovskite QDs.^55^ Lately, Zhang et al. synthesized 2D RPP (C_18_H_35_NH_3_)_2_SnBr_4_ through this method.^56^ The product was obtained by swiftly injecting a preheated SnBr_2_–TOP solution to an ODE solution containing quantitative OAc and oleylamine ligands at 180 °C protected by N_2_ gas. The reaction continued for 10 s, and then an ice bath was used to stop it. Finally, the product was obtained by adding hexane and then centrifuging. X‐ray diffraction (XRD) confirms a periodic diffraction pattern with a regular interval of 2.3° at small angles derived from the periodic 2D structure, similar to the previous reports.[qv: 15a,25a,52] This perovskite material with high fluorescence efficiency was used to make LEDs.^56^

### 2.2 2D Perovskite Thin Films

Appropriate thin film deposition technology is of great significance for obtaining high‐quality optoelectronic devices. Two common methods are spin coating and chemical vapor deposition. For obtaining perovskite thin films by spin coating, organic halide AX and bivalent metal halide BX_2_ (PbI_2_, PbBr_2_, or PbCl_2_) are dissolved in organic solvents to form precursor solutions, which are then spin casted or dropped onto different matrices and annealed to form perovskite thin films. It is very important to choose the appropriate processing time and temperature based on different precursor compositions for the needed crystallinity, phase state, and morphology of perovskite films.^57^ Some important research results are presented here.

**Figure 4:**
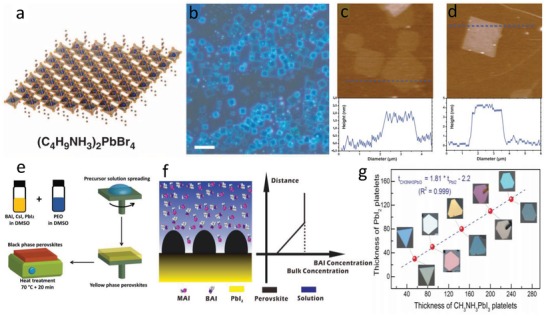
a) Structural illustration of a single‐layer (BA)_2_PbBr_4_ (blue balls for lead atoms, large orange balls for bromine atoms, red balls for nitrogen atoms, and small orange balls for carbon atoms; H atoms were omitted for clarity). b) Optical image of the 2D square sheets. Scale bar is 10 mm. c,d) AFM images and height profiles of several single/double layers with thickness of 1.6/3.4 nm (±0.2 nm). Reproduced with permission.[qv: 17c] Copyright 2015, American Association for the Advancement of Science (AAAS). e) Quasi‐2D perovskite/PEO composite thin film by spin coating followed by thermal annealing. Reproduced with permission.^60^ Copyright 2018, Wiley‐VCH. f) Proposed crystallization process and BAI concentration as a function of distance from the substrate. Reproduced with permission.^67^ Copyright 2018, American Chemical Society. g) Measured thicknesses and optical images of initial PbI_2_ nanoplatelets and corresponding CH_3_NH_3_PbI_3_ platelets. Reproduced with permission.^73^ Copyright 2014, Wiley‐VCH.

PEAI [(C_6_H_5_C_2_H_4_NH_3_)_2_I] and PbI_2_ were dissolved in DMF and then the solution was spin coated on a quartz substrate to form a (PEA)_2_PbI_4_ thin film.^58^ The film thickness varies from 3 to 100 nm, affected by the precursor concentration and the spin‐coating speed. Atomically thin uniform 2D square perovskite (BA)_2_PbBr_4_ was reported by Yang's group in 2015.[qv: 17c] A very dilute precursor solution was dropped onto a silica substrate and heated to dry under 75 °C. When a mixed solvent of DMF and chlorobenzene was used to dissolve BABr and PbBr_2_, the obtained products were thick and randomly distributed on the substrate. When acetonitrile was introduced to form a ternary mixed solvent, uniform square perovskite sheets grew on the substrate because of a faster evaporation (**Figure** **4**b). AFM image shows that the thicknesses of single and double layers were 1.6 and 3.4 nm, respectively (Figure 4c,d).

Butterfly‐shaped (BA)_2_PbI_4_ 2D perovskites with different sizes and thicknesses were synthesized by Fang et al. through growth control with temperature and mass ratio.^59^ A quasi‐2D perovskite (BA)_2_Cs*_n_*_−1_Pb*_n_*I_3_ *_n_*_+1_/PEO composite film (BA = benzyl ammonium, PEO = poly(ethylene oxide)) was used as light‐emitting layer to assemble efficient red light LEDs (Figure 4e).^60^ A lower temperature of 70 °C was enough for the phase transition of CsPbI_3_ perovskite from yellow phase to black phase, due to the confinement of inorganic layer of BA cation. More importantly, the introduction of PEO not only helps form nanoscale perovskites with smooth thin films due to its viscidity, but also promotes the charge transfer in the perovskite–PEO composite for good PLQYs because of its good ion conductivity.

It is well known that the PCE of perovskite solar cells depends heavily on the quality and morphology of thin films. Snaith's group introduced BA into 3D double‐cation perovskite FA_0.83_Cs_0.7_Pb(I_0.6_Br_0.4_)_3_. They obtained fully crystallized 2D/3D BA*_x_*(FA_0.83_Cs_0.7_)_1−_ *_x_*Pb(I_0.6_Br_0.4_)_3_ perovskite films by annealing as‐cast precursor films in air for 80 min at 175 °C. The presence of BA not only accelerated and evolved the high crystallinity of thin films, but also induced the change of lattice parameters of the 3D perovskite phase. The heterostructures between 2D and 3D perovskite phases passivated the interfacial grain boundary, thus inhibiting nonradiative recombination and achieving enhancement of performance and stability of perovskite solar cells.^61^ Very recently, Zhu's group fabricated a 2D perovskite (BA)_2_(Cs_0.02_MA_0.64_FA_0.34_)_4_PbI_6_ film with Cs^+^–MA^+^–FA^+^ triple cations by a simple spin coating at room temperature. Compared with 2D perovskite film with a monocation, the 2D triple‐cation perovskite has smoother, denser surface morphology, larger apparent grain size, and smaller grain boundary, leading to a longer carrier life and a higher conductivity.^62^ Recently, Gao's group reported a simple method for high quality of RPP films by incorporating dimethyl sulfoxide (DMSO) and MACl in the precursor solution, followed by one‐step spin coating and solvent annealing process. During crystallization, the synergistic effect of DMSO and MACl led to uniform morphology, good crystallinity, and reduced energy disorder.^63^

A novel hot‐coating technique has been proposed to achieve high‐quality RPP films with favorable orientation for charge transfer eventually.[qv: 35,45d,64] In order to obtain high‐quality films, Tsai et al. reported that 2D perovskite (BA)_2_MA*_n_*_−1_Pb*_n_*I_3_ *_n_*_+1_ single crystals were dissolved in DMF, and the solution was under continuous stirring for 30 min at 70 °C before the coating. Then, FTO/PEDOT:PSS substrates were preheated for 10 min from 30 to 150 °C, and the precursor solution was dropped on the hot substrate and spin coated at a speed of 5000 rpm for 20 s.^35^ The core of this technology is the precise control of the temperature of the substrates. From AFM and scanning electron microscopy (SEM) observations, the films obtained by hot coating not only have larger grains, leading to a more compact and uniform film, but also have lower pinhole density, compared with films obtained by room‐temperature coating. From the synchronous diffraction data, the main growth direction of perovskite is along (101) plane parallel to the *q_z_* direction.^35^ Recent studies of Sn‐based RPP films also suggest that the preferential orientation can be controlled by precursor solvents through hot‐coating method.[qv: 45d] Similar results were found in (BA)_2_(MA)_4_Pb_5_I_16_ RPP film, which also was highly oriented from DMF/DMSO mixtures by hot coating.^65^

A two‐step consecutive deposition^66^ was presented to grow quasi‐2D perovskite (BA)_2_(MA)*_n_*_−1_Pb*_n_*I_3_ *_n_*_+1_, a hierarchical structure with 2D perovskite on a 3D perovskite film.^67^ The growth mechanism of this hierarchical structure is a spatially limited nucleation of the nanosheets on 3D perovskite film, due to the respective concentration of BAI and MAI and their ratio. Especially, the vertical growth of perovskite nanosheets on a thin film is closely related to the concentration gradient of BAI as shown in Figure 4f.

A profound understanding of the growth mechanism is important for regulating the orientation of materials through precursors. Since a perfect band alignment naturally exists in the materials, this special structure can facilitate electron and hole transfer, which may further promote efficient emission and photovoltaic performance.^68^ The spin‐coating method is characterized by simple operation, low cost, and easy to form large area, but it is not easy to select the appropriate solvent that not only dissolves hybrid perovskite crystal precursors, but also has good wettability to the substrate. Molecular orientation degree and carrier mobility of the as‐prepared films are not high, and the thickness, uniformity, and surface morphology of the films are difficult to control, limiting the application range of the spin‐coating method.

Chemical vapor deposition is also widely applied to prepare 2D materials, such as graphene^69^ and transition metal sulfides.^70^ The materials obtained with this method have the advantages of higher crystallinity and fewer defects, but the yield is often low and the performance is not very reproducible, apparently not suitable for large scales. Liu et al. found that MAPbI_3−_ *_x_*Cl*_x_* perovskite thin film was much more uniform if prepared by a one‐step dual‐source (PbCl_2_ and MAI) vapor deposition than the one obtained through the solution process.^71^

In addition, aerosol‐assisted chemical vapor deposition has also been used to prepare perovskite films.^72^ Two‐step vapor deposition was used to equip MAPbI_3_ perovskites.^73^ First, with van der Waals force epitaxial growth, lead halide nanoplatelets were achieved on the muscovite mica; then, a gas–solid heterogeneous reaction was employed to convert the grown nanoplatelets to perovskites with methylammonium halide molecules. The lateral dimension was controlled from 5 to 10 µm. Figure 4g presents the relationship between CH_3_NH_3_I and PbI_2_ platelets—the perovskite platelet thickness was achieved by adjusting the thickness of the relevant lead halide platelets. Similar work has been reported by Shi's group. They demonstrated that weak van der Waals force played an important role in the growth of large‐sized single‐crystal 2D perovskites. Ionic crystals with delocalized bonds are more likely to form ultrathin structures than covalent compounds with localized bonds.^74^ By adjusting the pressure, temperature, and other conditions during the conversion process, it is expected to produce 2D mixed lead halide perovskites and realize a broad range adjustment of wavelength. In addition, there are other methods to prepare 2D perovskites, such as mechanical exfoliation^75^ and soft lithography.^76^ A good understanding of the experimental condition control on the material properties is essential to realize practical optoelectronic applications.

## 3 Diverse Properties of 2D Perovskites

The 2D RPPs are made of a series of alternately arranged inorganic and organic layers. They have a quantum well structure: the inorganic layer “well” is composed of metal halide, and the organic cation insulating layer acts as “barrier” to isolate the inorganic layer. The inorganic layers have large quantum confinement effect due to the small dielectric shielding effect from the organic cations, which confines the charge in the inorganic layer and is more conducive to charge recombination. Further, the number of stacked inorganic layers reflects the intensity of the quantum confinement effect. Single layer shows the strongest quantum confinement effect.

The change of each component of the structure will influence the properties. For example, the selection of cations is an important factor affecting the lattice orientation of inorganic layers and the number of inorganic layers is related to the reaction stoichiometric ratio. All these factors will change the physical and optical properties of 2D perovskites.

### 3.1 Excitons and Electronic Structure Properties

The exciton binding energy (*E*_b_) and bandgap of 2D perovskites are more significantly affected by dielectric and quantum confinement effects than those of 3D perovskites.^77^ In general, the dielectric constant of organic layers is much smaller than that of inorganic layers. In this case, the Coulomb interaction between the electron and the hole will be stronger because of the small shielding effect. So, the exciton binding energy of 2D perovskites is almost five times as high as that of 3D analogues.^78^ The bandgap (*E*_g_) and exciton binding energy (*E*_b_) of (quasi‐) 2D organic–inorganic halide perovskites are summarized in **Table** **2**.

**Table 2:** Summary of bandgap (*E*_g_) and exciton binding energy (*E*_b_) for (quasi‐) 2D organic–inorganic halide perovskites

Perovskite material	Bandgap *E*_g_ [eV]	Exciton binding energy *E*_b_ [meV]
(BA)_2_MA*_n_*_−1_Pb*_n_*I_3_ *_n_*_+1_ (*n* = 1–4)		
(BA)_2_PbI_4_	1.96–2.55[qv: 15a,25,44a,79]	380^80^, 290^81^
(BA)_2_MAPb_2_I_7_	1.99[qv: 15a], 2.17[qv: 25a]	170–270^80^, ^81^
(BA)_2_MA_2_Pb_3_I_10_	1.85[qv: 15a], 2.03[qv: 25a]	220^80^, 130^81^
(BA)_2_MA_3_Pb_4_I_13_	1.56[qv: 15a], 1.91[qv: 25a]	220^80^
(BA)_2_PbBr_4_	2.53[qv: 79b]	–
(BA)_2_FA_2_Pb_3_I_10_	1.51^82^	–
(BA)_2_MA*_n_*_−1_Ge*_n_*I_3_ *_n_*_+1_ (*n* = 1, 3, 4)		
(BA)_2_GeI_4_	1.74[qv: 79b]	–
(BA)_2_MA_2_Ge_3_I_10_	2.34^83^	–
(BA)_2_MA_3_Ge_4_I_13_	2.29^83^	–
(BA)_2_MA*_n_*_−1_Sn*_n_*I_3_ *_n_*_+1_ (*n* = 1, 3, 4)		
(BA)_2_SnI_4_	1.45^28^	–
(BA)_2_MA_2_Sn_3_I_10_	1.5[qv: 45d], 1.87^83^	–
(BA)_2_MA_3_Sn_4_I_13_	1.42[qv: 45d], 1.75^83^	–
(C_6_H_13_NH_3_)_2_MA*_n_*_−1_Pb*_n_*I_3_ *_n_*_+1_ (*n* = 1–4)		
(C_6_H_13_NH_3_)_2_PbI_4_	2.7^30^	310^84^
(C_6_H_13_NH_3_)_2_MAPb_2_I_7_	2.4^30^	–
(C_6_H_13_NH_3_)_2_MA_2_Pb_3_I_10_	2.17^30^	–
(C_6_H_13_NH_3_)_2_MA_3_Pb_4_I_13_	2.02^30^	–
(C_8_H_17_NH_3_)_2_PbBr_4_	3.1^85^	–
(C_8_H_17_NH_3_)_2_MA_2_Pb_3_Br_10_	2.26^51^	–
(C_8_H_17_NH_3_)_2_MA_2_Pb_3_I_10_	1.9^51^	–
(C_10_H_21_NH_3_)_2_PbI_4_	–	315[qv: 77b]
(C_6_H_5_CH_2_NH_3_)_2_PbI_4_	2.12–2.19[qv: 27a]	–
(PEA)_2_PbCl_4_	3.63^86^	–
(PEA)_2_MA*_n_*_−1_Pb*_n_*Br_3_ *_n_*_+1_ (*n* = 1–5)		
(PEA)_2_PbBr_4_	3.0^86^, ^87^	≥430[qv: 42a]
(PEA)_2_MA_2_Pb_3_Br_10_	2.5^88^	–
(PEA)_2_MA*_n_*_−1_Pb*_n_*I_3_ *_n_*_+1_ (*n* = 1–5)		
(PEA)_2_PbI_4_	2.3–3.14[qv: 16b,44a,77b,89]	220[qv: 42a]
(PEA)_2_MAPb_2_I_7_	2.32[qv: 44a], 2.19,^90^ 2.2[qv: 16b]	175[qv: 77b], 170[qv: 42a,44a]
(PEA)_2_MA_2_Pb_3_I_10_	2.10[qv: 15b], 2.04,^90^ 2.0[qv: 16b]	–
(PEA)_2_MA_2_Pb_5_I_16_	1.72[qv: 15b]	–
(PEA)_2_SnI_4_	1.97,^91^ 1.99^86^	160–190[qv: 42a]
(CH_3_CH_2_CH_2_NH_3_)_2_CsPb_2_I_7_	1.76^92^	–
(NH_3_C_8_H_16_NH_3_)PbI_4_	2.58^93^	–
(NH_3_C_8_H_16_NH_3_)MAPb_2_I_7_	2.15^93^	–
(NH_3_C_8_H_16_NH_3_)MA_2_Pb_3_I_10_	2.01^93^	–
(NH_3_C_8_H_16_NH_3_)MA_3_Pb_4_I_13_	1.9^93^	–

BA = C_4_H_9_NH_3_; PEA = C_6_H_5_C_2_H_4_NH_3_.

#### 3.1.1 Effect of Organic Layer

**Figure 5:**
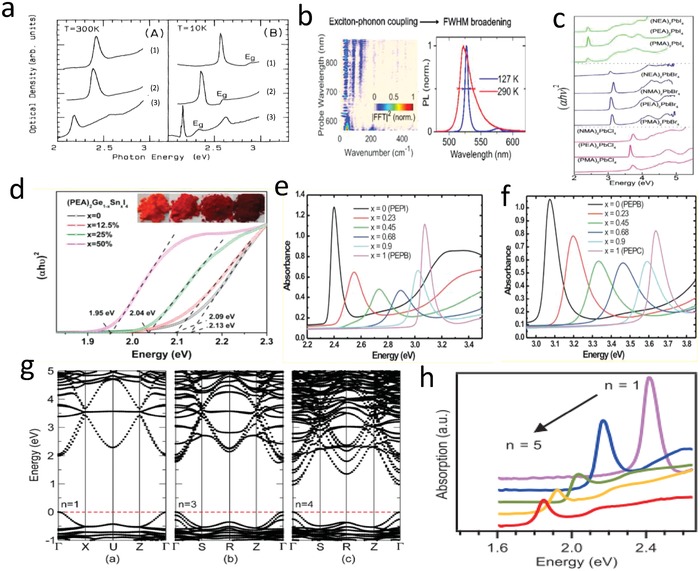
a) Absorption spectra at *T* = 300 and 10 K for 1) C_10_–PbI_4_, 2) PhE–PbI_4_, and 3) PhE–Pb_2_I_7_. Reproduced with permission.[qv: 77b] Copyright 1992, American Physical Society. b) Femtosecond vibrational spectroscopy and PL dependence on temperature. Reproduced with permission.^95^ Copyright 2017, American Chemical Society. c) UV–vis absorption spectra for 2D hybrid organic–inorganic perovskite films. Reproduced with permission.^26^ Copyright 2017, American Chemical Society. d) Tauc plots of (PEA)_2_Ge_1−_ *_x_*Sn*_x_*I_4_ (*x* = 0, 0.125, 0.25, 0.5). Photographs of the compounds with different Sn content are shown as inset. Reproduced with permission.^102^ Copyright 2018, American Chemical Society. e,f) Optical absorbance spectra of PhE–PbI_4(1−_ *_x_*_)_Br_4_ *_x_* and PhE–PbBr_4(1−_ *_x_*_)_Cl_4_ *_x_*. Reproduced with permission.[qv: 103] Copyright 2014, American Chemical Society. g) Electronic band structure of the polar configurations of selected (BA)_2_(MA)*_n_*_−1_Pb*_n_*I_3_ *_n_*_+1_ perovskites. Reproduced with permission.[qv: 25a] Copyright 2016, American Chemical Society. h) Absorption of the exfoliated crystals. Reproduced with permission.^80^ Copyright 2017, AAAS.

In 1990, Ishihara et al. reported the reflection spectra of (C*_n_*H_2_ *_n_*_+1_NH_3_)_2_PbI_4_ 2D perovskites with *n* = 4, 6, 8, 9, 10, and 12 in the region of 248–540 nm. The lattice spacing between the PbI_4_ layers increases as the number of carbon chain increases from 15.17 Å for *n* = 4 to 24.51 Å for *n* = 12. The *E*_b_ values of these compounds are nearly the same despite the different spacings. Among them, the *E*_b_ of (C_10_H_21_NH_3_)_2_PbI_4_ is 320 meV, which is higher than that of 3D lead iodide perovskites.^78^ Later, C_6_H_5_C_2_H_4_NH_3_ (PhE) with a greater dielectric constant due to its aromatic ring was introduced to replace decylammonium. Through the optical absorption spectra at *T* = 300 and 10 K (**Figure** **5**a),[qv: 77b] the ground‐state excitons of (C_10_H_21_NH_3_)_2_PbI_4_ and (PhE)_2_PbI_4_ are both measured at photon energy of 2.4 eV at room temperature. When the temperature decreases to 10 K, the exciton absorption peak suddenly became sharper; more importantly, the bandgap *E*_g_ of (PhE)_2_PbI_4_ is identified as 2.58 eV. By the formula *E*_b_ = *E*_g_ − (exciton peak energy),^78^, ^94^ *E*_b_ of (PhE)_2_PbI_4_ is 220 meV. As expected, a smaller exciton binding energy is obtained because of the larger dielectric confinement effects.

As described in Figure 5b, the fluorescence emission peak of (C_6_H_13_NH_3_)_2_PbI_4_ perovskite becomes sharper and more symmetrical at 127 K compared with that at 290 K. Through femtosecond vibrational spectroscopy, it is found that excitons of (BA)_2_PbI_4_ couple to phonons dominantly at 100 cm^−1^, while in (C_6_H_13_NH_3_)_2_PbI_4_ the wavenumbers are 88 and 137 cm^−1^. Therefore, Ni et al. confirmed that the selection of organic cations affected the exciton–phonon coupling.^95^ Moreover, strong exciton–phonon coupling may lead to a wider PL peak, which is undesirable for monochromatic LEDs. However, in some other applications, strong exciton–phonon coupling is desirable and may be beneficial for the white light emission^28^ and broadband short‐pulse lasers. The biexciton binding energy of (BA)_2_PbBr_4_ is found to be 60 meV and that of (C_6_H_13_NH_3_)_2_PbI_4_ is 44 meV. They are relatively larger values compared to other semiconductors. This is so because the value of biexciton binding energy depends on the gap energy difference between “well” and “barrier” in the quantum well structure.^96^ Recently, Sanvitto's group reported that biexciton also influenced exciton confinement and spectral response, in terms of affecting the out‐of‐plane exciton–photon interaction.^97^ Different organic cations cause different bandgap energies and lead to different biexciton binding energies.

Yan et al. reported that replacing MA with FA in (BA)_2_(MA)*_n_*_−1_Pb*_n_*I_3_ *_n_*_+1_ perovskites not only effectively reduced the bandgap of the 2D perovskites, but also improved their ambient stability.^82^ Particularly, the bandgap of (BA)_2_(FA)_2_Pb_3_I_10_ film is only 1.51 eV, which is much smaller than that of (BA)_2_(MA)_2_Pb_3_I_10_ (1.89 eV)[qv: 15a] and contributes to a good PCE of 6.88%. Quarti et al. proved that the electronic properties of the 2D perovskites were influenced by the length of the organic alkyl chain, and a longer chain led to an increase in the bandgap.^98^ Theoretical calculation of (C_6_H_13_NH_3_)_2_PbI_4_ and (C_12_H_25_NH_3_)_2_PbI_4_ indicates that this effect is caused by the distortion of the PbI_6_ octahedral structure due to the long alkyl chains. So, the size of organic cations plays an important role in adjusting the inorganic layer structure, thus leading to the regulation of the bandgap.[qv: 4b,27b,99] According to a recent report,^26^ the length of the alkyl chain between the aromatic ring and the ammonium group, rather than the number of aromatic rings, is vital in the bandgap of 2D perovskites containing aromatic cations (Figure 5c). In addition to the chain length and dielectric constant of cations discussed earlier, perovskite phase transition can also affect the structure and optical properties. Through in situ high‐pressure XRD, the shift of exciton bandgap of (BA)_2_PbI_4_ resulted from the change of Pb—I bond length and Pb—I—Pb bond angle derived from the pressure‐induced phase transition has been studied.[qv: 24b] The influence of structure phase transition of (C*_m_*H_2_ *_m_*_+1_NH_3_)_2_PbI_4_ (*m* = 4, 8, 9, 10, and 12) perovskites on bandgap was also observed between 235 and 310 K.^78^

#### 3.1.2 Effect of Inorganic Layer

Unlike conventional semiconductors, where their valence bands consist of p orbitals and conduction bands consist of s orbitals, the valence bands of 2D and 3D perovskites are mainly composed of p orbitals of halogens hybridized with the s orbitals of metals, while the conduction bands are emphatically made of the p orbitals of metals. In lead iodide–based perovskites, the valence band is relevant to the orbitals of I 5p and Pb 6s, and Pb 6p orbitals for conduction band.[qv: 44b] Therefore, both metal substitution and halogen doping can affect the bandgap of perovskites to achieve the desired properties.

2D perovskites with Sn and Ge are known to have smaller bandgaps than Pb‐based perovskites.[qv: 15d,38,52,83,100] For example, the bandgap of PEA_2_SnI_4_ is 2.19 eV, while that of PEA_2_PbI_4_ is 2.62 eV.^101^ The binding energies of excitons have also been reported to decrease from 230 to 160–190 meV for these two 2D perovskites.^101^ Recently, Zeng's group obtained the bandgap of BA_2_MI_4_ (M = Ge, Sn, and Pb) by theoretical calculations. The bandgaps of BA_2_GeI_4_, BA_2_SnI_4_, and BA_2_PbI_4_ are 1.74, 1.45, and 1.96 eV, respectively. BA_2_GeI_4_ is more affected than BA_2_SnI_4_ and BA_2_PbI_4_ by the distorted MI_6_ octahedra, which resulted from the reduced coordination symmetry around the cations by unbonded lone pair electrons.^83^ A series of mixed Ge–Sn halide–based 2D perovskites (PEA)_2_Ge_1−_ *_x_*Sn*_x_*I_4_ were synthesized by Han's group.^102^ It can be seen from Figure 5d that the bandgap reduces with the increase of Sn component. When *x* = 0.5, the smallest bandgap is 1.95 eV. A partial substitution of Sn not only reduces the bandgap, but also improves the conductivity, and the improvement of moisture stability of (PEA)_2_Ge_0.5_Sn_0.5_I_4_ is caused by the addition of PEA with hydrophobic groups, which is more helpful as a light‐absorbing material in solar cells.

The bandgap of 2D perovskites can also be changed by halide substitution. Replacing iodide with bromide and chloride will increase the bandgap of perovskite, because the maximum value of valence band (p orbitals) is lowered by the introduction of relatively high electronegative elements.[qv: 15d,16a,36,52,103] 2D perovskites with mixed halide such as (PEA)_2_PbZ_4(1−_ *_x_*_)_Y_4_ *_x_*, where Z and Y stand for I, Br, or Cl, have been reported.^103^ From the optical absorbance spectra shown in Figure 5e,f, strong absorption peaks are observed with narrow bandwidths, at 2.4 eV (I‐only), 3.1 eV (Br‐only), and 3.7 eV (Cl‐only), respectively, corresponding to a previous report.[qv: 2b] The absorption bands come from exciton formed by the transition from the Pb^2+^ 6s orbital to the Pb^2+^ 6p orbital, and the continuous regulation of bandgap is therefore realized. Compared with the single halide perovskites, the mixed halide perovskites have inhomogeneous broader absorption peak due to the disordered distribution of halides in inorganic layers.^104^ Recently, the same halide regulation of (PEA)_2_PbX_4_ was reported by Zhang's group with a highest PLQY of 46.5% for (PEA)_2_PbBr_4_.[qv: 15d]

The bandgap of perovskites can also be tuned through different number of inorganic layers.[qv: 25a,80,83,93,105] In the series of (BA)_2_(MA)*_n_*_−1_Pb*_n_*I_3_ *_n_*_+1_ perovskites, the optical absorption band energies are 2.43 eV (*n* = 1), 2.17 eV (*n* = 2), 2.03 eV (*n* = 3), 1.91 eV (*n* = 4), and 1.50 eV (*n* = ∞, it actually becomes MAPbI_3_). The bandgap decreases with the increase of *n* value depending on the stoichiometric ratio, which is attributed to the reduction of dielectric and quantum confinement effects.[qv: 25a] The corresponding fluorescence emission wavelengths also have a redshift with the increase of *n*. The (BA)_2_(MA)*_n_*_−1_Pb*_n_*I_3_ *_n_*_+1_ 2D perovskites are all semiconductors, with a clear direct bandgap shown in Figure 5g, where the valence band is mainly composed of I 5p and a small number of Pb 6s, while the conduction band is composed of Pb 6p orbital. The consistent results of exfoliated crystals (BA)_2_(MA)*_n_*_−1_Pb*_n_*I_3_ *_n_*_+1_ are also reported,[qv: 80] and the bandgap is in the range of 2.42 eV (*n* = 1) to 1.85 eV (*n* = 5) (Figure 5h). Importantly, the exciton binding energy decreases from 380 meV (*n* = 1) to an average 220 meV (*n* ≥ 2), which also results from the quantum confinement effects. Peng et al. reported that the bandgap of (PEA)_2_MA*_n_*_−1_Pb*_n_*I_3_ *_n_*_+1_ single crystal reduced continuously from 2.4 eV (*n* = 1) to 2.2 eV (*n* = 2) and 2.0 eV (*n* = 3), resembling other 2D perovskites.[qv: 16b]

### 3.2 Luminescence

#### 3.2.1 Narrow Emission

**Figure 6:**
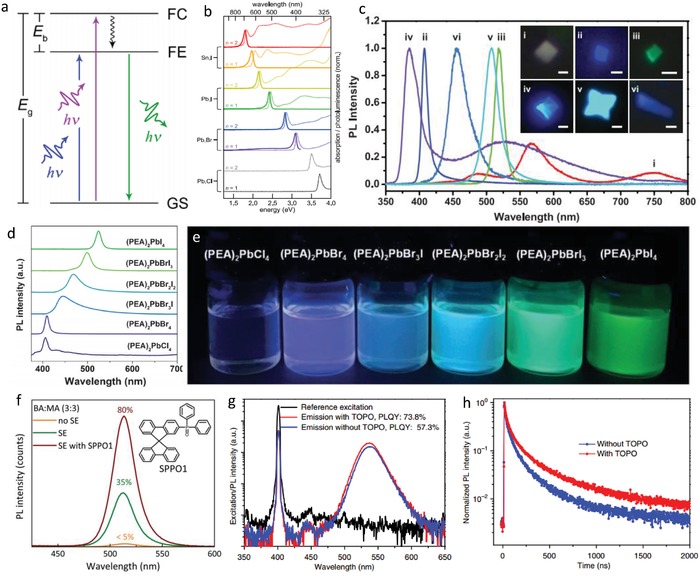
a) Energy‐level diagram of the typical excitonic and band‐to‐band transitions of 2D lead halide perovskites (FC = free carrier; FE = free exciton; GS = ground state). Colored arrows indicate absorption or PL, and the black arrow represents nonradiative relaxation. Reproduced with permission.^106^ Copyright 2018, American Chemical Society. b) Solution‐phase absorption (dotted lines) and PL (solid lines) spectra for *n* = 1 and *n* = 2 nanoplatelets in toluene, highlighting the changes that occur when the halide (X) changes from Cl to Br to I and when the metal changes from Pb to Sn. Reproduced with permission.^52^ Copyright 2016, American Chemical Society. c) PL of different 2D hybrid perovskites and the corresponding optical PL images. Scale bars are 2 mm for (i–v) and 10 mm for (vi). Reproduced with permission.[qv: 17c] Copyright 2015, AAAS. d) PL spectra of (PEA)_2_PbX_4_ NSs (X = Cl, Br, I) with different compositions. e) Photograph of solutions of (PEA)_2_PbX_4_ NSs with different composition under the irradiation of a 365 nm UV lamp. Reproduced with permission.[qv: 15d] Copyright 2017, Wiley‐VCH. f) PL spectra under excitation of 330 nm for the quasi‐2D compound with equimolar BA:MA ratio, without and with solvent evaporation, and with solvent evaporation in the presence of SPPO1. Reproduced with permission.^107^ Copyright 2017, Royal Society of Chemistry. g,h) PLQY and time‐resolved photoluminescence of the (PEA)_2_FA_2_Pb_3_Br_10_ perovskite films with and without TOPO passivation. Reproduced with permission.^108^ Copyright 2018, Springer Nature.

The recombination of free exciton is the source of narrow emission and small Stokes shift about (001) 2D lead halide perovskites. Stimulated by light, electrons transit from the ground state to the excited state, leaving holes in the ground state, and then the recombination of free exciton releases energy in the form of fluorescence, as shown in **Figure** **6**a.^106^ The merits of tunable color in the visible range and high PLQY of 2D perovskites are demonstrated. The luminescence with different wavelengths is realized through the regulation of metals (Pb and Sn) and halogens (Cl, Br, I) of L_2_[ABX_3_]*_n_*_−1_BX_4_ (*n* = 1 and 2) perovskites as depicted in Figure 6b.^52^ By replacing bromine with iodine of lead‐based perovskites, the emission peak moves to the direction of lower energy from 3.08 to 2.41 eV for *n* = 1, and from 2.82 to 2.16 eV for *n* = 2. By replacing Pb with Sn, the emission peak shifts to much lower energy of 1.97 (*n* = 1) and 1.80 eV (*n* = 2). 2D metal halide perovskites (BA)_2_PbX_4_ with high PLQY and adjusted band edge emission were reported by Dou et al.,[qv: 17c] as demonstrated in Figure 6c. Zhang's group reported tunable emission of ultrathin monolayer (PEA)_2_PbX_4_ 2D perovskites with halogen substitution.[qv: 15d] It can be seen from Figure 6d that the emission peak of (PEA)_2_PbI_4_ is located at 524.0 nm with a full width at half maximum (FWHM) of 14.7 nm. When the proportion of Br increases, the emission peak gradually shifts to blue, until (PEA)_2_PbBr_4_ forms with a highest PLQY of 46.5% at 409.1 nm and a narrow FWHM of 10.6 nm. Figure 6e shows the color change of (PEA)_2_PbX_4_ from violet to blue and finally green, under a 365 nm light excitation.

The PLQY of 2D perovskites is generally low due to the phase impurity and traps on the film surface from the solution process. In order to improve the luminescence efficiency, some surface modifications have been carried out. Bolink's group reported an impressive PLQY of (BA)_2_(MA)_4_Pb_5_Br_16_ thin film at 515 nm exceeding 80% with a molar ratio of 3:3 between BA and MA through the introduction of an electron donor SPPO1 (Figure 6f).^107^ The surface defects are effectively passivated to reduce the nonradiative recombination, so the radiative efficiency is greatly improved. Another example is to coat the surface of quasi‐2D PEA_2_(FA)*_n_*_−1_Pb*_n_*Br_3_ *_n_*_+1_ (*n* ≥ 2) perovskite film with trioctylphosphine oxide (TOPO).^108^ According to the report, all of the PEA_2_(FA)*_n_*_−1_Pb*_n_*Br_3_ *_n_*_+1_ perovskites have two emission peaks: a stronger green emission centered at about 532 nm from larger *n* phase and a weaker blue emission located at ≈440 nm from the *n* = 2 phase. Among them, the highest PLQY of 57.3% is from PEA_2_(FA)_2_Pb_3_Br_10_ (*n* = 3), which can be greatly improved to 73.8% with TOPO passivation. Moreover, the fluorescence decay time is also extended from 0.17 to 2 µs, as shown in Figure 6g,h.^108^

#### 3.2.2 Broad Emission

Different from the (001) 2D perovskites with a narrow emission, the corrugated (110) perovskites show a broad emission that spans the entire visible region. According to the corrugation length, these structures can be defined as “*n* × *n*”, where *n* stands for the number of octahedra in one unit. In the present study, the most common structure is 2 × 2, as shown in Figure 1c. The corrugation lengths with “3 × 3”^109^ or “4 × 4”^110^ have also been achieved, although they are just rare. The first corrugated (110) perovskite with 3 × 3 structure is α‐(DMEN)PbBr_4_ prepared by the Kanatzidis's group.^109^ Such a large distorted structure results from the special “chelating effect” of hydrogen bond interactions. α‐[NH_3_(CH_2_)_5_NH_3_]SnI_4_ and α‐(HA)SnI_4_ with 4 × 4 structure are only two examples reported.

**Figure 7:**
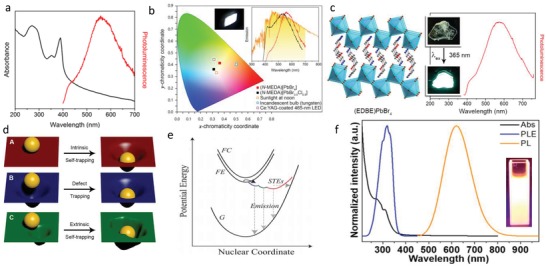
a) Absorption (black) and emission (red, excited at 380 nm) spectra for (*N*‐MEDA)[PbBr_4_]. b) Chromaticity coordinates (CIE) of white light emitters. Inset: photograph of luminescence from powders and solar spectrum (orange) with the visible region shaded in yellow and emission spectra of (*N*‐MEDA)[PbBr_4_] (red, excited at 380 nm) and (*N*‐MEDA)[PbBr_3.5_Cl_0.5_] (black, excited at 360 nm). Reproduced with permission.^11^ Copyright 2014, American Chemical Society. c) X‐ray crystal structure of the (110) perovskite (EDBE)PbBr_4_, and its emission spanning the entire visible spectrum. Inset: photographs of an (EDBE)PbBr_4_ crystal. Reproduced with permission.[qv: 111a] Copyright 2014, American Chemical Society. d) Self‐trapping (A), trapping at permanent defects (B), and self‐trapping influenced by permanent defects (C) represented by a ball interacting with a rubber sheet. Reproduced with permission.^106^ Copyright 2018, American Chemical Society. e) Schematic of the adiabatic potential energy curves of the ground state (G), free‐exciton state (FE), free‐carrier state (FC), and various excited states (STEs) in a configuration space. The horizontal dashed line shows possible nonradiative decay processes of the STEs. Reproduced with permission.[qv: 18a] Copyright 2016, American Chemical Society. f) Normalized absorption (Abs), PL excitation (PLE, monitored at 620 nm), and PL (excited by 365 nm) spectra of (OAm)_2_SnBr_4_ perovskite film. Inset: photograph of the colloidal suspension of (OAm)_2_SnBr_4_ perovskites under UV light. Reproduced with permission.^56^ Copyright 2019, American Chemical Society.

To date, 12 organic cations have been used to synthesize corrugated 2D perovskites with such a broad white light emission.[qv: 11,18c,28,109,111] The mechanism of this broadband luminescence is believed to be from the “self‐trapping states.” Karunadasa's group first reported the white light emission from three 2D perovskites: (110) perovskites of (*N*‐MEDA)PbBr_4_ (*N*‐MEDA = *N*′‐methylethane‐1,2‐diammonium) and (EDBE)PbBr_4_, and (001) perovskite of (EDBE)PbCl_4_.[qv: 11,111a] Under 380 nm excitation, (*N*‐MEDA)PbBr_4_ shows a wide emission covering the entire visible range with shoulder peak at 420 nm and an intense broad band at about 558 nm (**Figure** **7**a). (*N*‐MEDA)PbBr_4_ is used to make solid‐state lighting and shows a white light with CIE of (0.36, 0.41) and correlated color temperature (CCT) of 4669 K. In order to tune the emission chromaticity, (*N*‐MEDA)[PbBr_4−_ *_x_*Cl*_x_*] (*x* = 0 − 1.2) mixed halide perovskites were synthesized. (*N*‐MEDA)[PbBr_3.5_Cl_0.5_] results in a “cold” white light with CIE of (0.31, 0.36) and CCT of 6502 K, close to pure white light as seen in Figure 7b.^11^ White light–emitting 2D perovskite of (EDBE)PbBr_4_ was also prepared. Its PLQY was 9%, higher than that of (*N*‐MEDA)PbBr_4_.[qv: 111a] (EDBE)PbBr_4_ has a broad emission peak at 573 nm with an FWHM of 215 nm (Figure 7c). The emission has CIE of (0.39, 0.42) and CCT of 3990 K, corresponding to a “warm” white light. From the temperature‐dependent emission spectrum, when the temperature decreases from 300 to 150 K, the shoulder intensity becomes weaker and the FWHM is narrower.

The fluorescence spectrum hardly changes by changing the morphology and crystallinity, so the surface defects are not the cause of these wide emissions. According to the emission dependence on excitation intensity diagram, the PL intensity increases linearly with the increase of excitation intensity, and there is no PL saturation. Both indicate that the broadband emission is not from the permanent defects of the materials.[qv: 111a] Then, this wide fluorescence emission of (110) 2D perovskites is confirmed to be from the “excited‐state defects” formed from transient lattice distortions, which are induced by the coupling of photogenerated electrons/holes with the lattice. The intrinsic self‐trapping states of 2D perovskites can be explained by a model depicted in Figure 7d(A). The electron or hole is regarded as a hard ball. When the ball falls on the elastic sheet (soft lattice), the sheet is twisted, and then the sheet will return to its original state in the absence of the ball. This is different from the permanent defect trapping in that the distortion is already present before the ball drops onto the sheet, and the ball will sink with different indentation depths, as shown in Figure 7d(B). However, the extrinsic self‐trapping is related to lattice with local heterogeneity (Figure 7d(C)).^106^

Transient absorbance measurement is one of the most direct evidences for the exciton self‐trapping. Under the excitation of a near‐UV light, (*N*‐MEDA)PbBr_4_ shows a broad absorption in the range of visible spectrum, which is consistent with the formation of short‐lived, light‐induced defect states.[qv: 18a] In addition, for (*N*‐MEDA)PbBr_4_, the wavelength‐dependent PL shows that the onset time of broad emission is dependent on wavelength, and the decay time also shows the emission wavelength dependence due to the self‐trapped states.[qv: 18a] On the whole, these measurements prove the mechanism of the broad emission depicted in Figure 7e.[qv: 18a] After photon excitation, free excitons are formed in picoseconds, and then self‐trapped excitons formed by lattice distortion begin to generate broad emission, and the deeper the self‐trapped states, the lower the energy and the longer the PL wavelength. Zhang et al. reported that (OAm)_2_SnBr_4_ 2D perovskites emitted a wide orange light with a PLQY of 88%, which is the highest value among the known lead‐free 2D perovskites.^56^ Different from the white light emission with two peaks from the PbBr 2D perovskite, (OAm)_2_SnBr_4_ has only one PL peak located at 620 nm with an FWHM of 140 nm upon 365 nm excitation (Figure 7f). The emission is only from the exciton self‐trapping state, because the Sn^2+^ lone pair with higher chemical activity leads to stronger excited state structure distortion and coupling of photogenerated electrons/holes with the lattice of tin halide.^112^

The self‐trapping reflects the bulk properties of the lattice, so the broad PL emission can be regulated by changing the crystal structure through synthesis with various organic amine cations that are typically small, highly symmetric, or flexible ditopic, based on the in‐depth understanding about the relationship between the self‐trapping states and the crystal structure of perovskites.

### 3.3 Charge Carrier Transport

Solar cells and LEDs have different requirements for the charge transfer process. The charge transfer process is mainly determined by the interplay between carrier mobility (μ) and exciton binding energy (*E*_b_), so they play a guiding role in the design of efficient optoelectronic devices.^113^ Here, mobility refers to the velocity of charge carriers moving through conductive media under the electric field, and the binding energy of excitons is a representation of the strength of the binding force between an electron and a hole. Solar cells need fast charge separation, where both carrier radiative recombination and nonradiative recombination caused by defects need to be suppressed. In general, weak exciton binding and fast carrier mobility are required. To some extent, high mobility can reduce the contact time between the carrier and the trap, thus speeding up the escape rate from the shallow trap. However, in most cases, high mobility will actually speed up the trapping, so an appropriate mobility value is very important. LEDs ask for effective charge injection and radiative recombination, so nonradiative recombination resulting from defects should be avoided. For luminescent materials, strong exciton binding energy and low mobility increase the radiative recombination rate. However, in LED devices, low mobility can make charge injection unbalanced, and leads to charge accumulation, so that electrons accumulate at one side and holes at the other side of the device, resulting in lower device efficiency. A profound review about the design and construction of heterogeneous structures to improve the efficiency of charge transfer in semiconductor optoelectronic devices has been presented by Sargent's group.^113^ It is indisputable that charge transfer, which plays a governing role in different optoelectronic devices, is a key to the device design and needs careful study.^114^ In quasi‐2D perovskites with the existence of multiple phases, charge transfer is still controversial.

**Figure 8:**
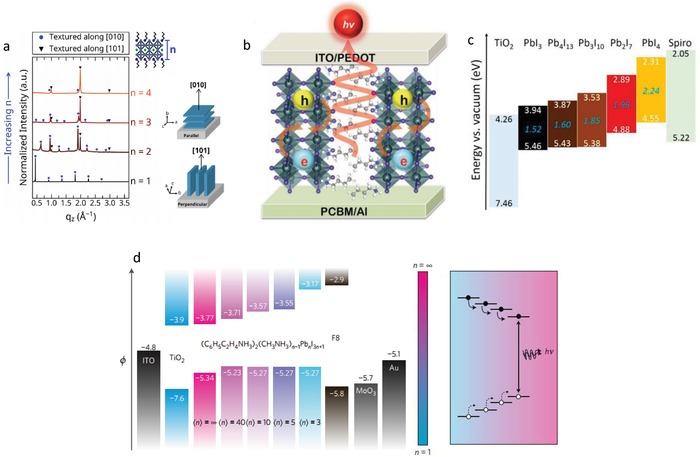
a) Specular X‐ray diffraction spectra of (BA)_2_(MA)*_n_*_−1_Pb*_n_*I_3_ *_n_*_+1_ for *n* = 1–4, illustrating an increase in the fraction of crystals textured along [101] with increasing *n* (left). Schematic of parallel and perpendicular texturing of lead iodide sheets (blue layers) along with their respective crystallographic axes (right). Reproduced with permission.^115^ Copyright 2018, American Chemical Society. b) Schematic illustration of the charge injection/recombination process in oriented film. Reproduced with permission.^116^ Copyright 2018, Wiley‐VCH. c) Comparative band energy diagram of (BA)_2_(MA)*_n_*_−1_Pb*_n_*I_3_ *_n_*_+1_ perovskite compounds. Reproduced with permission.[qv: 15a] Copyright 2015, American Chemical Society. d) Electronic energy levels of perovskites with different *n* values, combined with the band structure of ITO, TiO_2_, F8, MoO_3_, and the Au electrode. φ is the electrode potential. The arrows represent the carrier transfer processes. Reproduced with permission.[qv: 15b] Copyright 2016, Springer Nature.

In order to achieve an efficient charge transfer, the thin film preparation has to be improved. Recently, an orderly aligned orientation of (BA)_2_(MA)_3_Pb_4_I_13_ 2D perovskite was achieved through cation‐induced recrystallization process (CIRP).^36^ Compared with the random orientation without the CIRP treatment, the cations under design are evenly distributed, so the width of the quantum well is narrowed, which promotes the separation of charges and thus reduces the charge accumulation. This 2D perovskite is applied in a TiO_2_/Al_2_O_3_/NiO/C framework for solar cells, showing a fine PCE of 8.2%. The XRD of the film of (BA)_2_(MA)*_n_*_−1_Pb*_n_*I_3_ *_n_*_+1_ (*n* = 1–4) shows that the texture of crystal changes gradually, and the proportion of [101] textured domains increases, as the thickness *n* of lead iodide layer increases (**Figure** **8**a). Different from the lead iodide layer in the monolayer (*n* = 1) compound, which is preferentially arranged parallel to the substrate, it is almost completely perpendicular in the *n* = 4 compound.^115^ The same controlled orientation of (BA)_2_(MA)*_n_*_−1_Pb*_n_*I_3_ *_n_*_+1_ film is used to assemble LEDs (Figure 8b). Making inorganic layers perpendicular to the substrate, electrons and holes can be injected and transported to the deeper center of the film, rather than across the barrier of organic cations, compared with inorganic layers that are parallel to the substrate, thus improving the probability of radiative recombination.^116^

The complex multiphase distribution in quasi‐2D perovskites has caused a controversy of electronic energy band pattern in different optoelectronic devices. For solar cells based on 2D perovskites, the type‐II band alignment is always used (Figure 8c). It is described that both the conduction band and valence band have higher energies with a smaller number of *n*, compared with those of larger *n* phase of perovskites. Thus, the electrons transfer from the smaller *n* to the larger *n* domains, while the holes move in the opposite direction. Such a separation of electrons and holes enables efficient solar cells.[qv: 15a] However, the type‐I band alignment is proposed in the LEDs based on quasi‐2D perovskites (Figure 8d). The conduction band energy is lower when the *n* increases, while the valence band energy is almost the same. In this way, generated electrons and holes can be concentrated into the high‐*n* region where charge radiative recombination can be achieved. At the same time, it also effectively inhibits the nonradiative recombination, thus achieving LEDs with high efficiencies.[qv: 15b] In the complex multiphase distribution of quasi‐2D perovskites, the distribution ratio of each phase is often affected by the synthesis process in different laboratories, which may lead to the low reproducibility on the synthesis and structure of materials. This could be the source of the controversy for different device applications.^117^

## 4 Optoelectronic Applications

### 4.1 LEDs

Recently, organic–inorganic hybrid perovskites have been used in LEDs due to their highly efficient PL and wide color modulation in the visible and near‐infrared ranges. In addition to the above‐mentioned characteristics, (quasi‐) 2D perovskites show fascinating prospects in LED application with the large exciton binding energy. From the point of view of fabrication, layered 2D perovskites have good film processability with excellent optical properties. The published (quasi‐) 2D perovskite–based LEDs are summarized in **Table** **3**.

**Table 3:** (Quasi‐) 2D perovskite–based LEDs and their performances

Year	Emitting material	Device structure	EL [nm]	EQE_max_ [%]	CE_max_ [cd A^−1^]	*L*_max_ [cd m^−2^]	Ref.
1994	(PEA)_2_PbI_4_	ITO/EM/OXD7/Mg/Ag	520	–	–	10 000	118
2016	(PEA)_2_PbBr_4_	ITO/PEDOT:PSS/EM/TPBi/Al/Ca	410	0.04	–	–	87
2016	(PEA)_2_MA_4_Pb_5_I_16_	ITO/TiO_2_/EM/F8/MoO_3_/Au	≈760	8.80	–	–	[qv: 15b]
2016	(PBA)CsPbBr	ITO/PEIE–ZnO/EM/TFB/MoO*_x_*/Al	491	0.015	–	186	119
2016	(BA)_2_(MA)_2_Pb_3_I_10_ (BA)_2_(MA)_4_Pb_5_Br_16_ (BA)_2_(MA)_2_Pb_3_Br_7_Cl_3_	ITO/PEDOT:PSS/poly‐TPD/EM/TPBi/LiF/Al	700 523 468	2.29 1.01 0.01	0.10 3.48 0.006	214 2246 21	120
2016	NFPI_7_ NFPI_6_Br	ITO/PEIE–ZnO/EM/TFB/MoO*_x_*/Au	786 763	9.60 11.70	–	–	121
2016	MAPbBr_3_ (*n* = 7–10) MAPbBr_3_ (*n* = 5) MAPbBr_3_ (*n* = 3)	ITO/PEDOT:PSS/PVK/EM/TPBi/LiF/Al	520 492 456	2.31 0.233 0.024	8.10 0.28 0.049	1115 8.5 ≈1	[qv: 17b]
2016	POEA (30%):MAPbBr_3_ POEA (10%):MAPbBr_3_	ITO/PEDOT:PSS/EM/TPBi/Ba/Al	506/520 524	2.82 0.31	8.23 1.21	64.2 2146.11	122
2016	MAPbBr_3_:(PEA)_2_PbBr_4_ (1:6)	ITO/Buf‐HIL/EM/TPBi/Al	510–520	–	4.90	2935	123
2016	(PMA)_2_PbI_4_	ITO/PEDOT:PSS/EM/Bphen/Al	526	0.05	–	9	[qv: 89b]
2017	NCPI_6_Cl	ITO/PEIE–ZnO/EM/TFB/MoO*_x_*/Au	689	3.70	–	440	124
2017	(BA)_2_(MA)_4_Pb_5_Br_16_	ITO/PEDOT:PSS/EM/BmPyPhB/Ba/Ag	515	–	3.3	1000	107
2017	PEA_2_MA_4_Pb_5_Br_16_	ITO/PEDOT:PSS/EM/TPBi/LiF/Al	526	7.40	–	8400	125
2017	(EA)_2_(MA)*_n_*_−1_Pb*_n_*Br_3_ *_n_*_+1_	ITO/PEDOT:PSS/EM/TmPyPB/CsF/Al	473/485	2.60	–	200	126
2017	PEABr (80%):CsPbBr_3_	ITO/PEDOT:PSS/EM/TPBi/Ca/Al	514	1.97	6.16	9957	127
2017	BABr(20):MAPbBr_3_	ITO/PVK/EM/TPBi/LiF/Al	513	9.30	17.1	–	128
	BAI(20):MAPbI_3_	ITO/poly‐TPD/EM/TPBi/LiF/Al	748	10.4	0.09	–	
2017	PbS–(BA)_2_PbI_4_	ITO/TiO_2_/EM/F8/MoO_3_/Ag	1280	2.06	–	–	[qv: 79c]
2017	FAPbBr_3_	ITO/PEDOT:PSS/poly‐TPD/EM + PMMA/3TPYMB/LiF/Al	530	2.00	8.22	4425	129
2017	(PEA)_2_SnI_4_	ITO/PEDOT:PSS/EM/F8/LiF/Al	618	–	0.003	0.15	[qv: 45b]
2018	(BA)_2_(MA)_4_Pb_5_I_16_	ITO/PEDOT/EM/PCBM/Al	744	0.50	–	–	116
2018	(NMA)_2_Cs*_n_*_−1_Pb*_n_*I_3_ *_n_*_+1_	ITO/PEIE–ZnO/EM/TFB/MoO*_x_*/Au	694	7.30	–	732	130
2018	(PEA)_2_(FA)_2_Pb_3_Br_10_	ITO/PEDOT:PSS/EM/TOPO–TPBi/LiF/Al	532	14.36	62.4	9120	108
2018	(OA)_2_(FA)*_n_*_−1_Pb*_n_*Br_3_ *_n_*_+1_	ITO/PEDOT:PSS/EM/PO‐T2T/Ca/Al	530	13.40	57.6	34 480	131
2018	NFPI7	ITO/PEIE–ZnO/EM/TFB/MoO*_x_*/Au	≈790	12.7	–	–	132
2018	PEO:(BA)_2_(Cs)*_n_*_−1_Pb*_n_*I_3_ *_n_*_+1_	ITO/PEDOT:PSS/poly‐TPD/EM/BCP/LiF/Al	680	6.23	1.74	1392	60
2018	NF_0.93_C_0.07_PI_7_	ITO/PEIE–ZnO/EM/TFB/MoO*_x_*/Au	783	7.80	–	–	133
2018	(OA)_2_((FA)_2_)*_m_*_−1_Pb*_m_*Br_3_ *_m_*_+1_	ITO/LiF/EM/PO‐T2T/Ca/Al	540	5.00	22.9	≈2000	85
2018	PEABr:CsPbBr_3_	ITO/poly‐TPD/EM/TPBi/LiF/Al	510	15.5	49.1	7000	134
2018	3BBAI:MAPb(I/Cl)_3_	ITO/PTAA/EM/PC_61_BM/Cr/Au	760	3.85	–	–	135
2018	(NMA)_2_(FA)Pb_2_I_7_	ITO/PEIE–MZO/EM/TFB–PFO/MoO*_x_*/Au	≈795	20.1	–	–	136
2018	(BIZ)_2_(FA)*_n_*_−1_Pb*_n_*Br_3_ *_n_*_+1_	ITO/PVK/EM/TmPyPB/LiF/Al	≈535	7.70	–	30 000	137
2018	PA_2_CsPb_2_I_7_–CsPb(Br,Cl)_3_	ITO/PEDOT:PSS/PA_2_CsPb_2_I_7_/BIPO/poly‐TPD/PC_61_BM/CsPb(Br,Cl)_3_/TPBi/LiF/Al	693/493	0.22	–	–	92
2018	BA_2_Cs*_n_*_−1_Pb*_n_*Br_3_ *_n_*_+1_	ITO/PEDOT:PSS/EM/TPBi/Ca/Al	514	8.42	25.1	33 532	138
2018	(PEA)_2_PbCl_2_Br_2_	ITO/PEDOT:PSS/EM/TPBi/Ca/Al	425/500	–	–	70	139
2018	(PEA)_2_MA*_n_*_−1_Pb*_n_*Br_3_ *_n_*_+1_	ITO/PEDOT:PSS/EM/TPBi/Ca/Al	524	2.50	8.63	59 213	140
2018	(NMA)_2_PbBr_4_:FABr	ITO/TFB/PVK/EM/TPBi/LiF/Al	514	14.9	46.8	2056	141
2018	(TFA)_2_MA*_n_*_−1_Pb*_n_*Br_3_ *_n_*_+1_	ITO/PEDOT:PSS/PVK/EM/TPBi/LiF/Al	532	–	0.3	1200	142
2018	(OAm)_2_SnBr_4_	ITO/PEI–ZnO/EM/TCTA/MoO_3_/Au	625	0.1	0.029	350	56
2018	BA_2_Cs*_n_*_−1_Pb*_n_*(Br/Cl)_3_ *_n_*_+1_ BA_2_Cs*_n_*_−1_Pb*_n_*Br_3_ *_n_*_+1_ BA_2_Cs*_n_*_−1_Pb*_n_*(Br/I)_3_ *_n_*_+1_	ITO/PEDOT:PSS/PVK/EM/TPBi/Al	487 506 649	6.2 10.1 0.13	– 23.3 –	3340 3810 18.5	143
2019	BA_2_MA_3_Pb_4_Br_13_	ITO/PEDOT:PSS/EM/TPBi/LiF/Al	525	–	5.01	2819	144
2019	CsPbCl_0.9_Br_2.1_:PEABr	ITO/PEDOT:PSS/EM/TPBi/LiF/Al	480	5.7	6.1	3780	145

In order to achieve higher efficiency in LEDs, maximized radiative recombination is desired, while nonradiative recombination should be suppressed, which requires the regulation of basic material properties, such as defect state density, carrier mobility, and exciton binding energy. Quasi‐2D perovskites have large exciton binding energy, so electrons and holes can stay in a limited region for a period longer than decay time, providing a greater possibility for the radiative recombination (the radiative recombination rate depends on the overlapping ratio of wave functions of electrons and holes).^113^

It is well known that quasi‐2D perovskites are usually multiphase structures, and the phase impurity and disordered materials increase the likelihood to trap carriers, causing nonradiative recombination, thereby reducing emission efficiency. Thus, the precise regulation of components is crucial for effective radiation. However, the defects of 3D perovskites are caused by the halide vacancies in the material, or the surface dangling bonds made by the falling off of the surface ligands.^146^ In addition, the perovskite film obtained through the solution process, whether 3D or 2D perovskites, is treated at a low temperature, meaning that there is a great possibility to form surface defects.^113^ Therefore, improving the crystallinity of thin films is an important way to reduce defects. In addition to quenching temperature, the quasi‐2D perovskite films obtained by the solution process have a smaller crystal size than 3D perovskite films, which will increase the defect concentration on the surface and grain boundary of the films and the defects become the centers of nonradiative recombination, thereby reducing the emission efficiency. In addition, high surface area and porosity will also provide a greater probability of oxygen or water penetration. Recent reports have confirmed that 2D perovskites have a lower defect trap density due to the presence of ligands (organic amine cation) compared with 3D perovskites.^122^ In 3D perovskite LEDs and solar cells, surface passivation and doping are extensively studied as effective ways to reduce surface defect density. However, for quasi‐2D perovskite, these reports are relatively few. Stability is also an important indicator of device performance. The reported (quasi‐) 2D perovskites' electroluminescent lifetimes are listed in **Table** **4**.

**Table 4:** The operating stability of (quasi‐) 2D perovskite–based LEDs

2D perovskite material	EL [nm]	Operating voltage [V]	Current [mA cm^−2^]	Lifetime [h]	Atmosphere	Ref.
(NMA)_2_CsPb_2_I_6_Cl	689	–	10	5 (0.5 EQE)	–	124
(BA)_2_(MA)_3_Pb_4_I_13_	733	2	385.7	14 (0.5 radiance)	–	116
(BA)_2_(Cs)*_n_*_−1_Pb*_n_*I_3_ *_n_*_+1_	680	3.5	–	4 (0.8 EL intensity)	Air	60
(NMA)_2_FA_0.93_Cs_0.07_Pb_2_I_7_	783	–	10	31 (0.5 EQE)	N_2_	133
3BBAI:MAPb(I/Cl)_3_	760	–	200	95.88 (0.5 EQE)	Air	135
(NMA)_2_(FA)Pb_2_I_7_	795	–	0.1	46 (0.5 EL intensity)	Air	136
(PEA)_2_(MA)_2_Pb_3_Br_10_	510	–	10	7 (0.7 EL intensity)	N_2_	88
CsPbCl_0.9_Br_2.1_:PEABr	480	4.4	–	10 min (0.5 EQE)	–	145

Era et al. fabricated LEDs using (PEA)_2_PbI_4_ layered perovskite with a device structure of ITO/perovskite/OXD7/Mg/Ag in 1994.^118^ The strong electroluminescence (EL) peak at 520 nm was observed at liquid nitrogen temperature, and the maximum brightness of the device was up to 10 000 cd m^−2^. The effective EL is attributed to the introduction of OXD7, which not only acts as an appropriate electron transport layer, but also acts as a barrier layer to confine the holes in the emitting layer. This heterostructure provides an approach to achieve efficient LEDs. Sargent's group prepared LEDs with efficient multilayered quasi‐2D perovskite PEA_2_(MA)*_n_*_−1_Pb*_n_*I_3_ *_n_*_+1_. The device structure is ITO/TiO_2_/perovskite/F8/MoO_3_/Au, where TiO_2_ and F8 [poly(9,9′‐dioctylfluorence)] are electron and hole injection layers, respectively, as shown in Figure 8d. The LEDs with PEA_2_(CH_3_NH_3_)_4_Pb_5_I_16_ perovskite display a high external quantum efficiency (EQE) of 8.8% in the near‐infrared region, and the maximum radiance is 80 W sr^−1^ m^−2^ when the perovskite film is 200 nm. The good performance resulted from the efficient accumulation and recombination of electrons and holes occurring at the lowest bandgap of the multiphase quasi‐2D perovskites.[qv: 15b]

**Figure 9:**
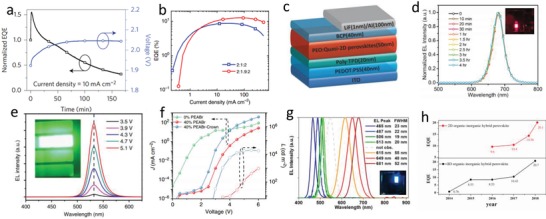
a) Stability data for a NFPI_7_ EL device tested at a constant current density of 10 mA cm^−2^. Reproduced with permission.^121^ Copyright 2016, Springer Nature. b) EQE versus current density. For the 2:1.9:2 multiple quantum well LEDs, a peak EQE of 12.7% is achieved at a current density of 80 mA cm^−2^. The EQE of the 2:1.9:2 device remains ≈10% at 500 mA cm^−2^ due to a significantly suppressed EQE roll‐off. Reproduced with permission.^132^ Copyright 2018, Springer Nature. c) Schematic of LED device structure. d) Electroluminescence spectral stability under 3.5 V continuous voltage operation; insets: photographs of devices at 4 V. Reproduced with permission.^60^ Copyright 2018, Wiley‐VCH. e) Typical EL spectra of (PEA)_2_FA_2_Pb_3_Br_10_‐based LEDs under different voltage biases. Inset shows the electroluminescence image of LEDs. Reproduced with permission.^108^ Copyright 2018, Springer Nature. f) *J*–*V*–*L* data and current efficiency of devices based on CsPbBr_3_ perovskite films with the introduction of 0% PEABr, 40% PEABr, and 40% PEABr‐crown. Reproduced with permission.^134^ Copyright 2018, Springer Nature. g) Normalized EL spectra of the CsPb(Br/Y)_3_ RPP devices at the turn‐on voltage; inset: photographs of the blue light LEDs. Reproduced with permission.^143^ Copyright 2018, American Chemical Society. h) Development trend of EQE of 2D and 3D organic–inorganic hybrid perovskite LEDs.

A series of LEDs with perovskite films that contain 1‐naphthylmethylamine iodide (NMAI), FAI/FABr, and PbI_2_ with a molar ratio of 2:1:2 was reported by Huang's group.^121^ A wide range of EL was achieved by adjusting the proportion of halogen components in the precursor solution, and the highest EQE of up to 11.7% at 763 nm with a radiance of 82 W sr^−1^ m^−2^ was achieved by (NMA)_2_Pb_2_I_6_Br (NFPI_6_Br) perovskite. Good device performance resulted from the complete surface coverage of the film, which reduces defects and leakage current, thereby suppressing nonradiative recombination. More importantly, the LEDs show improved lifetime due to the device's high efficiency and the perovskite film's superior stability. As shown in **Figure** **9**a, after 2 h continuous working at a current density of 10 mA cm^−2^, the EQE only decreases to half of its initial value. In order to reduce the efficiency roll‐off at high current density, Huang's group tuned the QW width by increasing the proportion of FA cation (the molar ratio of NMAI, FAI, and PbI_2_ changed from 2:1:2 to 2:1.9:2 in the precursors). The formation of wider QWs is proved by a 5.6 nm redshift of the PL peak. The wider QWs also suppress the luminescence quenching, so the EQE of LEDs is further improved to 12.7%. Additionally, the efficiency roll‐off is greatly reduced, and the efficiency is still maintained at about 10% under a current density of 500 mA cm^−2^ (Figure 9b). The device has a highest radiance of 254 W sr^−1^ m^−2^ in solution‐processed near‐infrared LEDs.^132^ The highest EQE of 20.1% of 2D perovskite LEDs to date in the near‐infrared range was reported by Friend's group,^136^ which is based on (NMA)_2_(FA)Pb_2_I_7_ 2D perovskite and poly‐HEMA (HEMA = 2‐hydroxyethyl methacrylate). This excellent EQE results from the ultrafast migration of excitons, which takes only ≈1 ps. It makes nonradiative recombination uncompetitive in dynamics and thus greatly suppresses bulk and interfacial nonradiative recombination.

Ma's group reported a series of efficient red light LEDs with emission peaks of 638, 664, and 680 nm based on quasi‐2D perovskite (BA)_2_Cs*_n_*_−1_Pb*_n_*I_3_ *_n_*_+1_/PEO composite with a device structure of ITO/PEDOT:PSS/poly‐TPD/perovskite/TPBi/LiF/Al (Figure 9c). The LEDs have a highest EQE of 6.23% and a brightness of 1293 cd m^−2^ at 680 nm emission peak, and show exceptional EL spectral stability under continuous operation (Figure 9d).^60^

Green light 2D perovskite LEDs based on (PEA)_2_(FA)*_n_*_−1_Pb*_n_*Br_3_ *_n_*_+1_ with EQE of 14.36% were reported by Yang et al.^108^ The reason for such a high EQE is the increased PL efficiency of the film due to less surface defect states caused by TOPO passivation; thus, nonradiative recombination at the surface and grain boundaries is reduced, as shown in Figure 6g. The EL spectra of (PEA)_2_(FA)_2_Pb_3_Br_10_‐based LEDs under different operating voltages are shown in Figure 9e. Different from the PL peak, there is only one single green EL peak located at 532 nm, while the blue light peak is not observed. The reason for this phenomenon is that the driving force for PL is the energy difference only, while the driving force of EL is the combination of energy difference and applied electric field, so most charges are injected into the smallest bandgap region and then recombine.

In order to achieve more efficient green light LEDs, the films are made of 2D organic–inorganic hybrid perovskite nanosheets and CsPbBr_3_ nanocrystals so as to provide an effective energy channel for the injection of excitons into the radiative recombination centers. However, there are still some problems with thin films, including crystallite distribution of CsPbBr_3_ nanocrystals and phase separation between the organic and inorganic phases.^134^, ^141^ Ban et al. demonstrated that the introduction of a crown molecule accurately controlled phase separation and improved film quality. Compared with CsPbBr_3_ LEDs, the leakage current of CsPbBr_3_ with 40% PEABr is lower and the turn‐on voltage of CsPbBr_3_ with 40% PEABr‐crown is further decreased. In addition, the current density and brightness are greatly improved (Figure 9f). So, the final EQE of these LEDs reaches 15.5% at 510 nm.[qv: 134]

Compared with the efficient near‐infrared, red, and green light LEDs, blue LEDs based on perovskites still have inferior performance. The ways to achieve blue light emission of LEDs are composition engineering and dimensional engineering. In 3D perovskites, Br is replaced by Cl to widen the perovskite bandgap to achieve blue light emission. In dimensional engineering, a reduced dimension enhances the quantum confinement effect, so the PL peak is shifted to blue light.

Recently, Cao's group reported an EQE of 5.7% for quasi‐2D perovskite LEDs with 480 nm blue emission.^145^ The introduction of PEABr into 3D perovskite CsPbCl_0.9_Br_2.1_ effectively passivates the surface trap of the film, where the trap density of perovskite film dramatically decreased from ≈4.1 × 10^17^ to 3.0 × 10^16^ cm^−3^ with the increase of PEABr ratio from 0 to 100%, and the PLQY increases from 0.15% to 27%. It can be seen that the effective inhibition of nonradiative combination is crucial to the PL efficiency of perovskites.

2D/3D mixed halide perovskites BA_2_Cs*_n_*_−1_Pb*_n_*(Br/X)_3_ *_n_*_+1_ (X = Cl, I) were reported to make LEDs with tunable color across the whole visible spectrum (Figure 9g).^143^ It is worth mentioning that the highest EQE for blue light at 486 nm is up to 6.2% with a luminance of 3340 cd m^−2^ at 8 V and the EQE reaches 10.1% at 506 nm. The first article on lead‐free 2D perovskite (PEA)_2_SnI*_x_*Br_4−_ *_x_*–based LEDs with a structure of ITO/PEDOT:PSS/EM/F8/LiF/Al was reported by Lanzetta et al. in 2017.[qv: 45b] Although the EQE of these LEDs is very low and the luminance is only 0.15 cd m^−2^, it indicates a possible way to fabricate LEDs from low‐dimensional lead‐free perovskites. Zhang's group recently reported improved 2D Sn‐based perovskite LEDs with an EQE of 0.1% and a maximum luminance of 350 cd m^−2^, which is the highest brightness of lead‐free perovskite LEDs to date and opens up their promising display application potentials.^56^

We summarized the annually reported highest efficiency of 2D and 3D organic–inorganic hybrid perovskite–based LEDs in recent years (Figure 9h). The EQE of 2D perovskite–based LEDs has a rapid development from 9.6% to 20.1% for green and near‐infrared emissions in just 3 years, and the current efficiency already approaches the level of 3D organic–inorganic hybrid perovskite–based LEDs. Apparently, 2D perovskites have a good prospect in LEDs.

### 4.2 Solar Cells

Today, 3D perovskites as light absorption layer for solar cells have reached a very good PCE as high as 24.2%.[qv: 7b] However, their sensitivity to the environment, especially moisture, is a major barrier to commercialization. Considerable efforts have been made to improve their stability.^147^ Compared to 3D perovskites, 2D perovskites have larger exciton binding energy and better stability in the ambient environment. However, 2D layered perovskites also bring some bad characteristics. First, the existence of long‐chain organic amine cation insulating layer and the unsatisfactory orientation of the inorganic layer structure will cause charge transfer problems, including charge accumulation and more charge recombination, so that the electrons and holes cannot be well separated.[qv: 22b,148] Second, as the number of layers decreases, the bandgap gradually widens, so the absorption of light is not ideal, thus resulting in a decrease in efficiency. Therefore, it is very important to achieve a balance between efficiency and stability by adjusting orientation and number of layers.

The first 2D layered perovskite solar cells based on (PEA)_2_(MA)_2_Pb_3_I_10_ were reported to have a PCE of 4.7%.^39^ Compared to MAPbI_3_, the 2D perovskite is more resistant to moisture, and due to the wider bandgap, the 2D structure is also suitable for the higher‐bandgap absorber in the dual‐absorber devices. Moreover, in terms of material optimization, 2D perovskite structure presents greater tunability at the molecular level. To date, a large number of 2D perovskite absorbers have been synthesized with significantly improved efficiencies.[qv: 35,64a,149] Sargent's group reported a PCE of 15.3% for quasi‐perovskite PEA_2_(CH_3_NH_3_)*_n_*_−1_Pb*_n_*I_3_ *_n_*_+1_ (*n* = 60). It shows an excellent stability with the efficiency remaining at about 13% after 2 weeks in a humid environment, while the efficiency of 3D MAPbI_3_ perovskite decreases from 16.6% to 4.3% in 3 days.[qv: 149d] (BA)_2_(MA)_2_PbI_3_‐based solar cell with PCE of 4.02% was obtained by Cao et al.[qv: 15a] Although the introduction of BA organic cation promotes resistance to moisture more than the 3D counterparts, it also causes out‐of‐plane charge transport inhibition. Notably, Tsai et al. overcame this disadvantage and achieved a vertical orientation of perovskite layers to the substrate by means of a hot‐casting deposition method. From the synchronous diffraction data, the main growth direction of perovskite is along (101) plane parallel to the *q_z_* direction. This unique orientation enables the photogenerated electrons and holes to move along the inorganic layer to the device's electrodes, respectively, avoiding the inhibition of organic layers. Such an efficient charge transport results in a PCE of 12.5% for (BA)_2_(MA)_3_Pb_4_I_13_ absorber solar cells.[qv: 35]

The introduction of 2D RPPs into 3D perovskites has been proved to guarantee a high efficiency and improved stability of the solar cells.[qv: 149d] For example, Liu's group reported a high PCE of 20.62% for 2D/3D heterostructure solar cells. The devices demonstrated significant long‐term ambient stability and worked for more than 2880 h when the efficiency dropped to 80% of the initial value without encapsulation.^150^ The introduction of BA changes the crystallization kinetics and controls the morphology of the film, resulting in larger particle size and improved film quality. The highest PCE (>22%) for 2D/3D perovskite solar cell was achieved by Grätzel's group, and the solar cells showed remarkable stability with 90% efficiency of 1000 h in moist air under simulated sunlight. The excellent performance comes from the formation of ultrathin, ultra‐hydrophobic, and highly uniform 2D (FEA)_2_PbI_4_ (FEA = phenylethylammonium) perovskite film casted on the 3D perovskite layer. The incorporation of (FEA)_2_PbI_4_ not only protects FAPbI_3_ film from the influence of moisture due to its hydrophobicity of fluoroarene, but also promotes the hole transfer from perovskite layer to spiro‐OMeTAD.^151^

Although 2D perovskite solar cells are at their start‐up stage, and the dielectric and quantum confinement effect plus carrier transport limit the PCE, they present excellent environmental stability far beyond 3D perovskites because of their unique layered structure. So, 2D perovskites lay the foundation for 2D/3D hybrid optoelectronic devices and will have a great potential for the solar cell commercialization.

## 5 Summary and Outlook

2D Ruddlesden–Popper perovskites have received widespread attention as promising materials for optoelectronic devices especially in recent years, due to their unique properties of large exciton binding energy, strong quantum confinement effect, and stability. Here, we reviewed the state‐of‐the‐art 2D perovskites, with their synthesis methods for powders and thin films, including single‐crystal growth, colloidal synthesis, spin coating, and vapor‐phase deposition, and analyzed the possible growth kinetics, various properties in optoelectronic devices, and applications in LEDs and solar cells.

Quasi‐2D perovskites have larger exciton binding energy, which is more conducive to radiative recombination. The EQE of LEDs based on quasi‐2D perovskites has reached 20.1% in near‐infrared emission, 15.5% in green light emission, and 6.2% in blue light emission. In order to achieve higher EQE and more stable LEDs, a few immediate issues need to be addressed. 1) The phase impurity and low‐temperature solution method for quasi‐2D perovskites often increase the defect density. So, the precise regulation of components and improvement of film quality are needed. 2) The poor transportation of charges in the organic amine spacing layers and the charge trapping in a surface trap limit the charge injection and reduce EQE. 3) The equilibrium between mobility and exciton binding energy should be further optimized for effective LEDs. Therefore, the design of heterostructures and efficient charge transfer channels are worth studying for quasi‐2D perovskites.

The solar cells based on 2D layered perovskites have demonstrated excellent PCE and superior stability. The highest PCE of 2D/3D perovskites has been over 22% and it can be maintained in humid air for more than 1000 h under simulated sunlight, while PCE has fallen by only 10%. It offers a route toward efficient and stable perovskite solar cells. However, there are still some problems to be solved before commercialization. 1) More efforts need to be made to fully understand their crystal growth mechanism and to further improve the quality and morphology of the films. 2) Similarly, the defects caused by the phase impurity of 2D perovskites will capture the charges and lead to nonradiative recombination, thus inhibiting the charge extraction. So, strictly following the stoichiometric ratio of the reaction to precisely control the value of *n* is desired. 3) In order to improve the charge transfer process in devices, thin films with out‐of‐plane orientation are desired and the technique of preparing controllable vertically oriented thin films needs to be improved. 4) Lead‐free perovskites have not yet achieved competitive device efficiencies, and their stability also needs to be improved.

## Conflict of Interest

The authors declare no conflict of interest.

## References

[1] a) Z. K. Tan , R. S. Moghaddam , M. L. Lai , P. Docampo , R. Higler , F. Deschler , M. Price , A. Sadhanala , L. M. Pazos , D. Credgington , Nat. Nanotechnol. 2014, 9, 687; 25086602 10.1038/nnano.2014.149

[2] a) K. Akihiro , T. Kenjiro , S. Yasuo , M. Tsutomu , J. Am. Chem. Soc. 2009, 131, 6050;19366264

[3] a) S. Chen , C. Teng , M. Zhang , Y. Li , D. Xie , G. Shi , Adv. Mater. 2016, 28, 5969; 27174465 10.1002/adma.201600468

[4] a) S. Adjokatse , H. H. Fang , M. A. Loi , Mater. Today 2017, 20, 413;

[5] a) G. Xing , N. Mathews , S. Sun , S. S. Lim , Y. M. Lam , M. Grätzel , S. Mhaisalkar , T. C. Sum , Science 2013, 342, 344; 24136965 10.1126/science.1243167

[6] a) Z. Xiao , Y. Yan , Adv. Energy Mater. 2017, 7, 1701136;

[7] a) W. S. Yang , B.‐W. Park , E. H. Jung , N. J. Jeon , Y. C. Kim , D. U. Lee , S. S. Shin , J. Seo , E. K. Kim , J. H. Noh , S. I. Seok , Science 2017, 356, 1376; 28663498 10.1126/science.aan2301

[8] V. M. Goldschmidt , Die Gesetze der Krystallochemie. Naturwissenschaften, 1926, 14, 477.

[9] L. Mao , C. C. Stoumpos , M. G. Kanatzidis , J. Am. Chem. Soc. 2019, 141, 1171. 30399319 10.1021/jacs.8b10851

[10] a) V. L. P. Guerra , P. Kovaricek , V. Vales , K. Drogowska , T. Verhagen , J. Vejpravova , L. Horak , A. Listorti , S. Colella , M. Kalbac , Nanoscale 2018, 10, 3198; 29379917 10.1039/c7nr07860a

[11] E. R. Dohner , E. T. Hoke , H. I. Karunadasa , J. Am. Chem. Soc. 2014, 136, 1718. 24422494 10.1021/ja411045r

[12] J. Hu , L. Yan , W. You , Adv. Mater. 2018, 30, 1802041. 10.1002/adma.201802041 30199108

[13] a) B. Saparov , D. B. Mitzi , Chem. Rev. 2016, 116, 4558; 27040120 10.1021/acs.chemrev.5b00715

[14] a) T. Kataoka , T. Kondo , R. Ito , S. Sasaki , K. Uchida , N. Miura , Phys. B Condens Matter. 1993, 184, 132; 10.1103/physrevb.47.2010 10006239

[15] a) D. H. Cao , C. C. Stoumpos , O. K. Farha , J. T. Hupp , M. G. Kanatzidis , J. Am. Chem. Soc. 2015, 137, 7843; 26020457 10.1021/jacs.5b03796

[16] a) N. Kitazawa , Mater. Sci. Eng. B 1997, 49, 233;

[17] a) K. Pradeesh , J. J. Baumberg , G. V. Prakash , Appl. Phys. Lett. 2009, 95, 033309;

[18] a) T. Hu , M. D. Smith , E. R. Dohner , M. J. Sher , X. Wu , M. T. Trinh , A. Fisher , J. Corbett , X. Y. Zhu , H. I. Karunadasa , J. Phys. Chem. Lett. 2016, 7, 2258; 27246299 10.1021/acs.jpclett.6b00793

[19] a) S. Sourisseau , N. Louvain , W. Bi , N. Mercier , D. Rondeau , F. Boucher , J.‐Y. Buzaré , C. Legein , Chem. Mater. 2009, 19, 600; 10.1021/ic070240g 17595074

[20] Y. I. Dolzhenko , T. Inabe , Y. Maruyama , Bull. Chem. Soc. Jpn. 1986, 59, 563.

[21] T. Ishihara , J. Takahashi , T. Goto , Solid State Commun. 1989, 69, 933.

[22] a) L. Etgar , Energy Environ. Sci. 2017, 11, 234;

[23] N. Kawano , M. Koshimizu , Y. Sun , N. Yahaba , Y. Fujimoto , T. Yanagida , K. Asai , J. Phys. Chem. C 2014, 118, 9101.

[24] a) K. Leng , I. Abdelwahab , I. Verzhbitskiy , M. Telychko , L. Chu , W. Fu , X. Chi , N. Guo , Z. Chen , Z. Chen , C. Zhang , Q.‐H. Xu , J. Lu , M. Chhowalla , G. Eda , K. P. Loh , Nat. Mater. 2018, 17, 908; 30202109 10.1038/s41563-018-0164-8

[25] a) C. C. Stoumpos , D. H. Cao , D. J. Clark , J. Young , J. M. Rondinelli , J. I. Jang , J. T. Hupp , M. G. Kanatzidis , Chem. Mater. 2016, 28, 2852;

[26] K.‐z. Du , Q. Tu , X. Zhang , Q. Han , J. Liu , S. Zauscher , D. B. Mitzi , Inorg. Chem. 2017, 56, 9291. 28749133 10.1021/acs.inorgchem.7b01094

[27] a) M. E. Kamminga , H.‐H. Fang , M. R. Filip , F. Giustino , J. Baas , G. R. Blake , M. A. Loi , T. T. M. Palstra , Chem. Mater. 2016, 28, 4554;

[28] K. Thirumal , W. K. Chong , W. Xie , R. Ganguly , S. K. Muduli , M. Sherburne , M. Asta , S. Mhaisalkar , T. C. Sum , H. S. Soo , N. Mathews , Chem. Mater. 2017, 29, 3947.

[29] X. Chen , H. Lu , Z. Li , Y. Zhai , P. F. Ndione , J. J. Berry , K. Zhu , Y. Yang , M. C. Beard , ACS Energy Lett. 2018, 3, 2273.

[30] K. Tanaka , T. Kondo , Sci. Technol. Adv. Mater. 2003, 4, 599.

[31] T. Ogawa , Y. Kanemitsu , Optical Properties of Low‐Dimensional Materials, World Scientific, Singapore 1995, 288.

[32] O. Nazarenko , M. R. Kotyrba , S. Yakunin , M. Aebli , G. Rainò , B. M. Benin , M. Wörle , M. V. Kovalenko , J. Am. Chem. Soc. 2018, 140, 3850. 29502407 10.1021/jacs.8b00194 PMC5867663

[33] A. Lemmerer , D. G. Billing , Crystengcom 2012, 14, 1954.

[34] Y. Tabuchi , K. Asai , M. Rikukawa , K. Sanui , K. Ishigure , J. Phys. Chem. Solids 2000, 61, 837.

[35] H. Tsai , W. Nie , J.‐C. Blancon , C. C. S. Toumpos , R. Asadpour , B. Harutyunyan , A. J. Neukirch , R. Verduzco , J. J. Crochet , S. Tretiak , L. Pedesseau , J. Even , M. A. Alam , G. Gupta , J. Lou , P. M. Ajayan , M. J. Bedzyk , M. G. Kanatzidis , A. D. Mohite , Nature 2016, 536, 312. 27383783 10.1038/nature18306

[36] H. Li , J. Lu , T. Zhang , Y. Shen , M. Wang , ACS Energy Lett. 2018, 3, 1815.

[37] a) B. Žekš , R. Blinc , R. Kind , Ferroelectrics 1978, 21, 2;

[38] D. B. Mitzi , Chem. Mater. 1996, 8, 791.

[39] I. C. Smith , E. T. Hoke , D. Solisibarra , M. D. Mcgehee , H. I. Karunadasa , Angew. Chem. 2015, 126, 11414.

[40] J. S. Manser , J. A. Christians , P. V. Kamat , Chem. Rev. 2016, 116, 12956. 27327168 10.1021/acs.chemrev.6b00136

[41] M. Safdari , P. H. Svensson , M. T. Hoang , I. Oh , L. Kloo , J. M. Gardner , J. Mater. Chem. A 2016, 4, 15638.

[42] a) C. G. Papavassiliou , Mol. Cryst. 1996, 286, 231;

[43] D. B. Mitzi , J. Solid State Chem. 1999, 145, 694.

[44] a) T. Ishihara , J. Lumin. 1994, 60, 269;

[45] a) C. R. Kagan , D. B. Mitzi , C. D. Dimitrakopoulos , Science 1999, 286, 945; 10542146 10.1126/science.286.5441.945

[46] D. B. Mitzi , C. A. Feild , W. T. A. Harrison , A. M. Guloy , Nature 1994, 369, 467.

[47] P. Cheng , T. Wu , J. Zhang , Y. Li , J. Liu , L. Jiang , X. Mao , R.‐F. Lu , W.‐Q. Deng , K. Han , J. Phys. Chem. Lett. 2017, 8, 4402. 28856895 10.1021/acs.jpclett.7b01985

[48] a) L. Protesescu , S. Yakunin , M. I. Bodnarchuk , F. Krieg , R. Caputo , C. H. Hendon , R. X. Yang , A. Walsh , M. V. Kovalenko , Nano Lett. 2016, 15, 3692; 10.1021/nl5048779 PMC4462997 25633588

[49] L. C. Schmidt , A. Pertegã , S. Gonzãlaz‐Carrero , O. Malinkiewicz , S. Agouram , G. M. Espallargas , H. J. Bolink , R. E. Galian , J. Pérez‐Prieto , J. Am. Chem. Soc. 2014, 136, 850. 24387158 10.1021/ja4109209

[50] J. A. Sichert , Y. Tong , N. Mutz , M. Vollmer , S. Fischer , K. Z. Milowska , R. García Cortadella , B. Nickel , C. Cardenas‐Daw , J. K. Stolarczyk , A. S. Urban , J. Feldmann , Nano Lett. 2015, 15, 6521. 26327242 10.1021/acs.nanolett.5b02985

[51] S. Aharon , L. Etgar , Nano Lett. 2016, 16, 3230. 27089497 10.1021/acs.nanolett.6b00665

[52] M. C. Weidman , M. Seitz , S. D. Stranks , W. A. Tisdale , ACS Nano 2016, 10, 7830. 27471862 10.1021/acsnano.6b03496

[53] Z. Yuan , C. Zhou , J. Messier , Y. Tian , Y. Shu , J. Wang , Y. Xin , B. Ma , Adv. Opt. Mater. 2016, 4, 2009.

[54] a) X. Zhang , Y. Zhang , L. Yan , C. Ji , H. Wu , Y. Wang , P. Wang , T. Zhang , Y. Wang , T. Cui , J. Zhao , W. W. Yu , J. Mater. Chem. A 2015, 3, 8501;

[55] a) M. Lu , X. Zhang , Y. Zhang , J. Guo , X. Shen , W. W. Yu , A. L. Rogach , Adv. Mater. 2018, 30, 1804691; 10.1002/adma.201804691 30306648

[56] X. Zhang , C. Wang , Y. Zhang , X. Zhang , S. Wang , M. Lu , H. Cui , S. V. Kershaw , W. W. Yu , A. L. Rogach , ACS Energy Lett. 2018, 242.

[57] J. Chen , L. Gan , F. Zhuge , H. Li , J. Song , H. Zeng , T. Zhai , Angew. Chem., Int. Ed. 2017, 56, 2390. 10.1002/anie.201611794 28097787

[58] K. Gauthron , J. S. Lauret , L. Doyennette , G. Lanty , A. Al Choueiry , S. J. Zhang , A. Brehier , L. Largeau , O. Mauguin , J. Bloch , E. Deleporte , Opt. Express 2010, 18, 5912. 20389609 10.1364/OE.18.005912

[59] C. Fang , J. Li , J. Wang , R. Chen , H. Wang , S. Lan , Y. Xuan , H. Luo , P. Fei , D. Li , Crystengcom 2018, 20, 6538.

[60] Y. Tian , C. Zhou , M. Worku , X. Wang , Y. Ling , H. Gao , Y. Zhou , Y. Miao , J. Guan , B. Ma , Adv. Mater. 2018, 30, 1707093. 10.1002/adma.201707093 29602181

[61] Z. Wang , Q. Lin , F. P. Chmiel , N. Sakai , L. M. Herz , H. J. Snaith , Nat. Energy 2017, 2, 17135.

[62] L. Gao , F. Zhang , X. Chen , C. Xiao , B. Larson , S. Dunfield , J. Berry , K. Zhu , Angew. Chem., Int. Ed. 2019, 58, 11737. 10.1002/anie.201905690 31218795

[63] J. Qing , X.‐K. Liu , M. Li , F. Liu , Z. Yuan , E. Tiukalova , Z. Yan , M. Duchamp , S. Chen , Y. Wang , S. Bai , J.‐M. Liu , H. J. Snaith , C.‐S. Lee , T. C. Sum , F. Gao , Adv. Energy Mater. 2018, 8, 1800185.

[64] a) X. Zhang , X. Ren , B. Liu , R. Munir , X. Zhu , D. Yang , J. Li , Y. Liu , D.‐M. Smilgies , R. Li , Z. Yang , T. Niu , X. Wang , A. Amassian , K. Zhao , S. Liu , Energy Environ. Sci. 2017, 10, 2095;

[65] C. M. M. Soe , W. Nie , C. C. Stoumpos , H. Tsai , J.‐C. Blancon , F. Liu , J. Even , T. J. Marks , A. D. Mohite , M. G. Kanatzidis , Adv. Energy Mater. 2018, 8, 1700979.

[66] T. M. Koh , V. Shanmugam , J. Schlipf , L. Oesinghaus , P. Müller‐Buschbaum , N. Ramakrishnan , V. Swamy , N. Mathews , P. P. Boix , S. G. Mhaisalkar , Adv. Mater. 2016, 28, 3653. 26990287 10.1002/adma.201506141

[67] L. Li , N. Zhou , Q. Chen , Q. Shang , Q. Zhang , X. Wang , H. Zhou , J. Phys. Chem. Lett. 2018, 9, 1124. 29432017 10.1021/acs.jpclett.7b03294

[68] J. Liu , J. Leng , K. Wu , J. Zhang , S. Jin , J. Am. Chem. Soc. 2017, 139, 1432. 28094931 10.1021/jacs.6b12581

[69] K. S. Novoselov , D. Jiang , F. Schedin , T. J. Booth , V. V. Khotkevich , S. V. Morozov , A. K. Geim , Proc. Natl. Acad. Sci. U.S.A. 2005, 102, 10451. 16027370 10.1073/pnas.0502848102 PMC1180777

[70] a) S. Balendhran , J. Z. Ou , M. Bhaskaran , S. Sriram , S. Ippolito , Z. Vasic , E. Kats , S. Bhargava , S. Zhuiykov , K. Kalantarzadeh , Nanoscale 2012, 4, 461; 22064926 10.1039/c1nr10803d

[71] M. Liu , M. B. Johnston , H. J. Snaith , Nature 2013, 501, 395. 24025775 10.1038/nature12509

[72] D. J. Lewis , P. O'Brien , Chem. Commun. 2014, 50, 6319. 10.1039/c4cc02592j 24799177

[73] S. T. Ha , X. Liu , Q. Zhang , D. Giovanni , T. C. Sum , Q. Xiong , Adv. Opt. Mater. 2014, 2, 838.

[74] Y. Wang , Y. Shi , G. Xin , L. Jie , S. Jian , Cryst. Growth Des. 2015, 15, 4741.

[75] W. Niu , A. Eiden , G. Vijaya Prakash , J. J. Baumberg , Appl. Phys. Lett. 2014, 104, 591.

[76] M. E. Kamminga , H.‐H. Fang , M. A. Loi , G. H. ten Brink , G. R. Blake , T. T. M. Palstra , J. E. ten Elshof , ACS Appl. Mater. Interfaces 2018, 10, 12878. 29578335 10.1021/acsami.8b02236 PMC5909174

[77] a) L. V. Keldysh , Sov. JETP. Lett. 1979, 29, 658;

[78] T. Ishihara , J. Takahashi , T. Goto , Phys. Rev. B Condens Matter. 1990, 42, 11099. 9995391 10.1103/physrevb.42.11099

[79] a) O. Yaffe , A. Chernikov , Z. M. Norman , Y. Zhong , A. Velauthapillai , A. van der Zande , J. S. Owen , T. F. Heinz , Phys. Rev. B 2015, 92, 045414;

[80] J. C. Blancon , H. Tsai , W. Nie , C. C. Stoumpos , L. Pedesseau , C. Katan , M. Kepenekian , C. M. Soe , K. Appavoo , M. Y. Sfeir , Science 2017, 355, 1288. 28280250 10.1126/science.aal4211

[81] X. Wu , M. T. Trinh , X. Y. Zhu , J. Phys. Chem. C 2015, 119, 14714.

[82] J. Yan , W. Fu , X. Zhang , J. Chen , W. Yang , W. Qiu , G. Wu , F. Liu , P. Heremans , H. Chen , Mater. Chem. Front. 2018, 2, 121.

[83] L. Ma , M.‐G. Ju , J. Dai , X. C. Zeng , Nanoscale 2018, 10, 11314. 29897093 10.1039/c8nr03589j

[84] K. Tanaka , F. Sano , T. Takahashi , T. Kondo , R. Ito , K. Ema , Solid State Commun. 2002, 122, 249.

[85] N. Yantara , A. Bruno , A. Iqbal , N. F. Jamaludin , C. Soci , S. Mhaisalkar , N. Mathews , Adv. Mater. 2018, 30, 1800818. 10.1002/adma.201800818 29971842

[86] G. C. Papavassiliou , I. B. Koutselas , Synth. Met. 1995, 71, 1713.

[87] D. Liang , Y. Peng , Y. Fu , M. J. Shearer , J. Zhang , J. Zhai , Y. Zhang , R. J. Hamers , T. L. Andrew , S. Jin , ACS Nano 2016, 10, 6897. 27336850 10.1021/acsnano.6b02683

[88] W. Bi , X. Huang , Y. Tang , H. Liu , P. Jia , K. Yu , Y. Hu , Z. Lou , F. Teng , Y. Hou , Org. Electron. 2018, 63, 216.

[89] a) J. Gebhardt , Y. Kim , A. M. Rappe , J. Phys. Chem. C 2017, 121, 6569;

[90] Q. Shang , Y. Wang , Y. Zhong , Y. Mi , L. Qin , Y. Zhao , X. Qui , X. Liu , Q. Zhang , J. Phys. Chem. Lett. 2017, 8, 4431. 28845670 10.1021/acs.jpclett.7b01857

[91] L. Lanzetta , J. M. Marin‐Beloqui , I. Sanchez‐Molina , D. Ding , S. A. Haque , ACS Energy Lett. 2017, 2, 1662.

[92] J. Mao , H. Lin , F. Ye , M. Qin , J. M. Burkhartsmeyer , H. Zhang , X. Lu , K. S. Wong , W. C. H. Choy , ACS Nano 2018, 12, 10486. 30222315 10.1021/acsnano.8b06196

[93] X. Li , J. Hoffman , W. Ke , M. Chen , H. Tsai , W. Nie , A. D. Mohite , M. Kepenekian , C. Katan , J. Even , M. R. Wasielewski , C. C. Stoumpos , M. G. Kanatzidis , J. Am. Chem. Soc. 2018, 140, 12226. 30169031 10.1021/jacs.8b07712

[94] T. Ishihara , X. Hong , J. Ding , A. V. Nurmikko , Surf. Sci. 1992, 267, 323.

[95] L. Ni , U. Huynh , A. Cheminal , T. H. Thomas , R. Shivanna , T. F. Hinrichsen , S. Ahmad , A. Sadhanala , A. Rao , ACS Nano 2017, 11, 10834. 29064668 10.1021/acsnano.7b03984

[96] Y. Kato , D. Ichii , K. Ohashi , H. Kunugita , K. Ema , K. Tanaka , T. Takahashi , T. Kondo , Solid State Commun. 2003, 128, 15.

[97] A. Fieramosca , L. De Marco , M. Passoni , L. Polimeno , A. Rizzo , B. L. T. Rosa , G. Cruciani , L. Dominici , M. De Giorgi , G. Gigli , L. C. Andreani , D. Gerace , D. Ballarini , D. Sanvitto , ACS Photonics 2018, 5, 4179.

[98] C. Quarti , N. Marchal , D. Beljonne , J. Phys. Chem. Lett. 2018, 9, 3416. 29870266 10.1021/acs.jpclett.8b01309

[99] M. R. Filip , G. E. Eperon , H. J. Snaith , F. Giustino , Nat. Commun. 2014, 5, 5757. 25502506 10.1038/ncomms6757

[100] C. C. Stoumpos , L. Mao , C. D. Malliakas , M. G. Kanatzidis , Inorg. Chem. 2017, 56, 56. 27997156 10.1021/acs.inorgchem.6b02764

[101] G. C. Papavassiliou , I. B. Koutselas , A. Terzis , M. H. Whangbo , Solid State Commun. 1994, 91, 695.

[102] P. Cheng , T. Wu , J. Liu , W.‐Q. Deng , K. Han , J. Phys. Chem. Lett. 2018, 9, 2518. 29699393 10.1021/acs.jpclett.8b00871

[103] G. Lanty , K. Jemli , Y. Wei , J. Leymarie , J. Even , J. S. Lauret , E. Deleporte , J. Phys. Chem. Lett. 2016, 5, 3958. 10.1021/jz502086e 26276477

[104] M. D. Smith , E. J. Crace , A. Jaffe , H. I. Karunadasa , Annu. Rev. Mater. Res. 2018, 48, annurev.

[105] Y. Zhang , R. Wang , Y. Li , Z. Wang , S. Hu , X. Yan , Y. Zhai , C. Zhang , C. Sheng , J. Phys. Chem. Lett. 2019, 10, 13. 30556395 10.1021/acs.jpclett.8b03458

[106] M. D. Smith , H. I. Karunadasa , Acc. Chem. Res. 2018, 51, 619. 29461806 10.1021/acs.accounts.7b00433

[107] M.‐G. La‐Placa , G. Longo , A. Babaei , L. Martinez‐Sarti , M. Sessolo , H. J. Bolink , Chem. Commun. 2017, 53, 8707. 10.1039/c7cc04149g 28722068

[108] X. Yang , X. Zhang , J. Deng , Z. Chu , J. Qi , J. Meng , P. Wang , L. Zhang , Z. Yin , J. You , Nat. Commun. 2018, 9, 570. 29422600 10.1038/s41467-018-02978-7 PMC5805756

[109] L. Mao , Y. Wu , C. C. Stoumpos , M. R. Wasielewski , M. G. Kanatzidis , J. Am. Chem. Soc. 2017, 139, 5210. 28306254 10.1021/jacs.7b01312

[110] a) J. Guan , Z. J. Tang , A. M. Guloy , Chem. Commun. 1999, 1833;

[111] a) E. R. Dohner , J. Adam , L. R. Bradshaw , H. I. Karunadasa , J. Am. Chem. Soc. 2014, 136, 13154; 25162937 10.1021/ja507086b

[112] H. Shi , D. Han , S. Chen , M.‐H. Du , Phys. Rev. Mater. 2019, 3, 034604.

[113] O. Voznyy , B. R. Sutherland , A. H. Ip , D. Zhitomirsky , E. H. Sargent , Nature Rev. Mater. 2017, 2, 17026.

[114] G. Xing , B. Wu , X. Wu , M. Li , B. Du , Q. Wei , J. Guo , E. K. Yeow , T. C. Sum , W. Huang , Nat. Commun. 2017, 8, 14558. 28239146 10.1038/ncomms14558 PMC5333353

[115] N. R. Venkatesan , J. G. Labram , M. L. Chabinyc , ACS Energy Lett. 2018, 3, 380.

[116] H. Tsai , W. Nie , J. C. Blancon , C. C. Stoumpos , M. M. S. Chan , J. Yoo , J. Crochet , S. Tretiak , J. Even , A. Sadhanala , Adv. Mater. 2018, 30, 1704217.

[117] Z. Chen , Y. Guo , E. Wertz , J. Shi , Adv. Mater. 2019, 31, 1803514. 10.1002/adma.201803514 30368915

[118] M. Era , S. Morimoto , T. Tsutsui , S. Saito , Appl. Phys. Lett. 1994, 65, 676.

[119] L. Cheng , Y. Cao , R. Ge , Y.‐Q. Wei , N.‐N. Wang , J.‐P. Wang , W. Huang , Chin. Chem. Lett. 2017, 28, 29.

[120] H. Hu , T. Salim , B. Chen , Y. M. Lam , Sci. Rep. 2016, 6, 33546. 27633084 10.1038/srep33546 PMC5025709

[121] N. Wang , L. Cheng , R. Ge , S. Zhang , Y. Miao , W. Zou , C. Yi , Y. Sun , Y. Cao , R. Yang , Y. Wei , Q. Guo , Y. Ke , M. Yu , Y. Jin , Y. Liu , Q. Ding , D. Di , L. Yang , G. Xing , H. Tian , C. Jin , F. Gao , R. H. Friend , J. Wang , W. Huang , Nat. Photonics 2016, 10, 699.

[122] Z. Chen , C. Zhang , X.‐F. Jiang , M. Liu , R. Xia , T. Shi , D. Chen , Q. Xue , Y.‐J. Zhao , S. Su , H.‐L. Yip , Y. Cao , Adv. Mater. 2017, 29, 1603157. 10.1002/adma.201603157 28000969

[123] J. Byun , H. Cho , C. Wolf , M. Jang , A. Sadhanala , R. H. Friend , H. Yang , T.‐W. Lee , Adv. Mater. 2016, 28, 7515. 27334788 10.1002/adma.201601369

[124] S. Zhang , C. Yi , N. Wang , Y. Sun , W. Zou , Y. Wei , Y. Cao , Y. Miao , R. Li , Y. Yin , Adv. Mater. 2017, 29, 1606600. 10.1002/adma.201606600 28417480

[125] L. N. Quan , Y. Zhao , G. D. A. Fp , R. P. Sabatini , G. Walters , O. Voznyy , R. Comin , Y. Li , J. Z. Fan , H. Tan , Nano Lett. 2017, 17, 3701. 28475344 10.1021/acs.nanolett.7b00976

[126] Q. Wang , J. Ren , X. Peng , X. Ji , X. Yang , ACS Appl. Mater. Interfaces 2017, 9, 29901. 28812341 10.1021/acsami.7b07458

[127] Y. F. Ng , S. A. Kulkarni , S. Parida , N. F. Jamaludin , N. Yantara , A. Bruno , C. Soci , S. Mhaisalkar , N. Mathews , Chem. Commun. 2017, 53, 12004. 10.1039/c7cc06615e 29053160

[128] Z. Xiao , R. A. Kerner , L. Zhao , N. L. Tran , K. M. Lee , T.‐W. Koh , G. D. Scholes , B. P. Rand , Nat. Photonics 2017, 11, 108.

[129] S. Kumar , J. Jagielski , N. Kallikounis , Y.‐H. Kim , C. Wolf , F. Jenny , T. Tian , C. J. Hofer , Y.‐C. Chiu , W. J. Stark , T.‐W. Lee , C.‐J. Shih , Nano Lett. 2017, 17, 5277. 28770603 10.1021/acs.nanolett.7b01544

[130] J. Chang , S. Zhang , N. Wang , Y. Sun , Y. Wei , R. Li , C. Yi , J. Wang , W. Huang , J. Phys. Chem. Lett. 2018, 9, 881. 29392948 10.1021/acs.jpclett.7b03417

[131] X. Y. Chin , A. Perumal , A. Bruno , N. Yantara , S. A. Veldhuis , L. Martínez‐Sarti , B. Chandran , V. Chirvony , A. S.‐Z. Lo , J. So , C. Soci , M. Grätzel , H. J. Bolink , N. Mathews , S. G. Mhaisalkar , Energy Environ. Sci. 2018, 11, 1770.

[132] Z. Wei , R. Li , S. Zhang , Y. Liu , N. Wang , C. Yu , Y. Miao , M. Xu , Q. Guo , D. Di , Nat. Commun. 2018, 9, 608. 29426896 10.1038/s41467-018-03049-7 PMC5807308

[133] M. Yang , N. Wang , S. Zhang , W. Zou , Y. He , Y. Wei , M. Xu , J. Wang , W. Huang , J. Phys. Chem. Lett. 2018, 9, 2038. 29620368 10.1021/acs.jpclett.8b00600

[134] M. Ban , Y. Zou , J. P. H. Rivett , Y. Yang , T. H. Thomas , Y. Tan , T. Song , X. Gao , D. Credington , F. Deschler , H. Sirringhaus , B. Sun , Nature Commun. 2018, 9, 3892. 30250032 10.1038/s41467-018-06425-5 PMC6155305

[135] R. Yang , R. Li , Y. Cao , Y. Wei , Y. Miao , W. L. Tan , X. Jiao , H. Chen , L. Zhang , Q. Chen , H. Zhang , W. Zou , Y. Wang , M. Yang , C. Yi , N. Wang , F. Gao , C. R. McNeill , T. Qin , J. Wang , W. Huang , Adv. Mater. 2018, 30, 1804771. 10.1002/adma.201804771 30345566

[136] B. Zhao , S. Bai , V. Kim , R. Lamboll , R. Shivanna , F. Auras , J. M. Richter , L. Yang , L. Dai , M. Alsari , X.‐J. She , L. Liang , J. Zhang , S. Lilliu , P. Gao , H. J. Snaith , J. Wang , N. C. Greenham , R. H. Friend , D. Di , Nat. Photonics 2018, 12, 783.

[137] M. Yu , C. Yi , N. Wang , L. Zhang , R. Zou , Y. Tong , H. Chen , Y. Cao , Y. He , Y. Wang , M. Xu , Y. Liu , Y. Jin , W. Huang , J. Wang , Adv. Opt. Mater. 2018, 7, 1801575.

[138] Z. Wang , F. Wang , W. Sun , R. Ni , S. Hu , J. Liu , B. Zhang , A. Alsaed , T. Hayat , Z. a. Tan , Adv. Funct. Mater. 2018, 28, 1804187.

[139] P. Cai , X. Wang , H. J. Seo , X. Yan , Appl. Phys. Lett. 2018, 112, 153901.

[140] Y. Han , S. Park , C. Kim , M. Lee , I. Hwang , Nanoscale 2018, 11, 3546. 10.1039/c8nr07361a 30565624

[141] T. Wu , Y. Yang , Y. Zou , Y. Wang , C. Wu , Y. Han , T. Song , Q. Zhang , X. Gao , B. Sun , Nanoscale 2018, 10, 19322. 30324959 10.1039/c8nr04896g

[142] G. Jia , Z.‐J. Shi , Y.‐D. Xia , Q. Wei , Y.‐H. Chen , G.‐C. Xing , W. Huang , Opt. Express 2018, 26, A66. 29402056 10.1364/OE.26.000A66

[143] P. Vashishtha , M. Ng , S. B. Shivarudraiah , J. E. Halpert , Chem. Mater. 2018, 31, 83.

[144] W. Zhang , X. Yan , W. Gao , J. Dong , R. Ma , L. Liu , M. Zhang , Org. Electron. 2019, 65, 56.

[145] Z. Li , Z. Chen , Y. Yang , Q. Xue , H.‐L. Yip , Y. Cao , Nat. Commun. 2019, 10, 1027. 30833581 10.1038/s41467-019-09011-5 PMC6399279

[146] X. Li , F. Cao , D. Yu , J. Chen , Z. Sun , Y. Shen , Y. Zhu , L. Wang , Y. Wei , Y. Wu , H. Zeng , Small 2017, 13, 1603996. 10.1002/smll.201603996 28067991

[147] a) F. Li , H. Wang , D. Kufer , L. Liang , W. Yu , E. Alarousu , C. Ma , Y. Li , Z. Liu , C. Liu , Adv. Mater. 2017, 29, 1602432; 10.1002/adma.201602432 28225207

[148] X. Zhang , G. Wu , S. Yang , W. Fu , Z. Zhang , C. Chen , W. Liu , J. Yan , W. Yang , H. Chen , Small 2017, 13, 1700611. 10.1002/smll.201700611 28692766

[149] a) W. Fu , J. Wang , L. Zuo , K. Gao , F. Liu , D. S. Ginger , A. K. Y. Jen , ACS Energy Lett. 2018, 3, 2086;

[150] T. Niu , J. Lu , M.‐C. Tang , D. Barrit , D.‐M. Smilgies , Z. Yang , J. Li , Y. Fan , T. Luo , I. McCulloch , A. Amassian , S. Liu , K. Zhao , Energy Environ. Sci. 2018, 11, 3358.

[151] Y. Liu , S. Akin , L. Pan , R. Uchida , N. Arora , J. V. Milic , A. Hinderhofer , F. Schreiber , A. R. Uhl , S. M. Zakeeruddin , A. Hagfeldt , M. I. Dar , M. Gratzel , Sci. Adv. 2019, 5, 2543. 10.1126/sciadv.aaw2543 PMC6555633 31187060

